# Mean performances, character associations and multi-environmental evaluation of chilli landraces in north western Himalayas

**DOI:** 10.1038/s41598-024-51348-5

**Published:** 2024-01-08

**Authors:** Thakur Narender Singh, A. K. Joshi, Amit Vikram, Nitin Yadav, Sakshi Prashar

**Affiliations:** Department of Vegetable Science, Dr. YS Parmar University of Horticulture and Forestry, Nauni Solan, Himachal Pradesh 173230 India

**Keywords:** Agricultural genetics, Genetic interaction, Heritable quantitative trait, Plant breeding, Plant genetics

## Abstract

Even though many varieties have been recommended across agro-climate zones of Himachal Pradesh, yet the information on stability is lacking in this State. Hence, the present investigation was carried out to identify high yielding stable genotypes among various pre-adapted landraces. The material consists of 20 chilli landraces including check i.e. DKC-8. The experiment was laid out in a RCBD. The data were recorded and analyzed to work out mean performances and the inferences were drawn for parameters of variability, correlation coefficients, path coefficients and stability analysis. As per mean performances, CS7 and CS9 were earliest in flowering, CS13 is earliest in days to ripe maturity, CS10 had highest plant height and CS9 had highest average fruit weight and ripe fruit yield plant^−1^. High PCV and GCV were recorded for ripe fruit yield plant^−1^. Heritability and genetic advance were recorded maximum for plant height in summer seasons and were recorded maximum for number of ripe fruits plant^−1^ in winter season. Correlation coefficients showed that number of ripe fruits plant^−1^ and average ripe fruit weight were positively and significantly correlated with ripe fruit yield plant^−1^. Path coefficient analysis in summer and winter seasons showed that average ripe fruit weight had the highest positive direct effect on ripe fruit yield plant^−1^. The pooled data over environments were analyzed to estimate the interaction effects between genotypes × environment. The mean sum of squares due to genotypes, environments and genotypes × environment interaction were significant for all the characteristics. CS1, CS3, CS6, CS10, CS13, CS15 were adapted to all environments, CS7 and CS9 were specifically adapted to favourable environment and CS2 was specifically adapted to unfavorable environment for 50% flowering, landraces CS1, CS2 and CS3were well adapted to all environments for ripe maturity whereas landraces CS6, CS10 and CS19 were well adapted to all environment for number of ripe fruit and ripe fruit yield.

## Introduction

Chilli (*Capsicum annuum* L.) popularly known as ‘*Lal Mirch’*, Hot pepper and pungent pepper is important Solanaceous crop grown throughout the world. It has originated from the wild and weedy species *Capsicum annuum var. minimum* distributed from the southern United States to northern South America^1^. The archaeological excavations at Tehuacan in Mexico have indicated the existence of chilli around 7000 B.C. prior to the advent of agriculture when it was used by man. It was introduced into southern part of India by the Portuguese in the sixteenth century^2^.India is the highest producer, consumer and exporter of chilli, accounting for nearly 33 per cent of the country’s total spice exports and a 16 per cent share of global spice trade. Andhra Pradesh is the leading producer of chilli in India, followed by Telangana and Madhya Pradesh. In Andhra Pradesh, chilli productivity is 46.57 q/ha whereas in Himachal Pradesh, chilli productivity is 11.9 q/ha^3^. These figures are alarmingly lesser to Andhra Pradesh average. In Himachal Pradesh, harsh winter, low yielding varieties and exorbitant cost of the hybrid seed coupled with enhanced input supply are major hurdles to the farmers for round the year cultivation of chilli in *ex-situ* condition. This indicates that there is need to increase average productivity of chilli in Himachal Pradesh by cultivating pre-adapted landraces because these landraces may prove are high yielding when grown *ex-situ*.

The main goal of the chilli breeding programme has been to develop varieties that can thrive in a wide range of environment conditions. As a consequence, determining the subsistence and degree of genotype × environment interaction, also identifying phenotypically stable landrace(s) with low genotype × environment interaction, is important. This requires the screening of promising landraces in a set of environmental conditions. It has been reported that the efficacy of various local landraces of a crop differs significantly from location to location and even more so, from season to season. The absence or presence of genotypes × environment interaction determines the average response of genotypes and high yield suggests that genotypes are ideal for general adaptation across a wide range of environments. Genotypes with high-stability are typically low-yielders and vice versa^2^. As a result, any crop improvement programme should aim in compensating these extremes. This is especially the case for vegetable crops, which are generally grown under a variety of agro-climatic, edaphic and management conditions. Before cultivars are released for commercial cultivation, it becomes imperative identify genotypes which are high yielding and performing consistently and uniformly across varied environments. The low productivity of chilli in India is due to the fact that open pollinated varieties cover the majority of the land (Pandit and Adhikary)^4^ There are several other constraints to good production, including unsuitable cultivars and hybrids, cultivar genetic drift, biotic and Abiotic stresses, home labour drifted and emergence of new diseases like bacterial wilt are the key bottleneck for achieving high production is a scarcity of improved varieties. Even though many varieties have been recommended across agro-climate zones of Himachal Pradesh, yet the information on stability is lacking in this State. Hence, the present investigation was carried out to identify high yielding stable genotypes among various pre-adapted landraces with the following objectives-

(i) To study the extent and magnitude of genetic variability among chilli landraces of Shillai, District Sirmour.
(ii) To find out character association between different horticultural and yield traits.
(iii) To identify phenotypically stable genotype(s) for ameliorating chilli productivity.

## Materials and methods

### Experimental site

**Figure 1:**
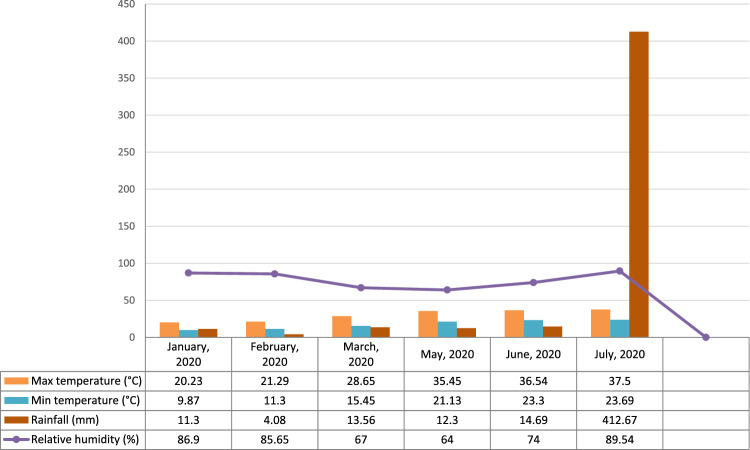
Meteorological data of summer season 2020.

**Figure 2:**
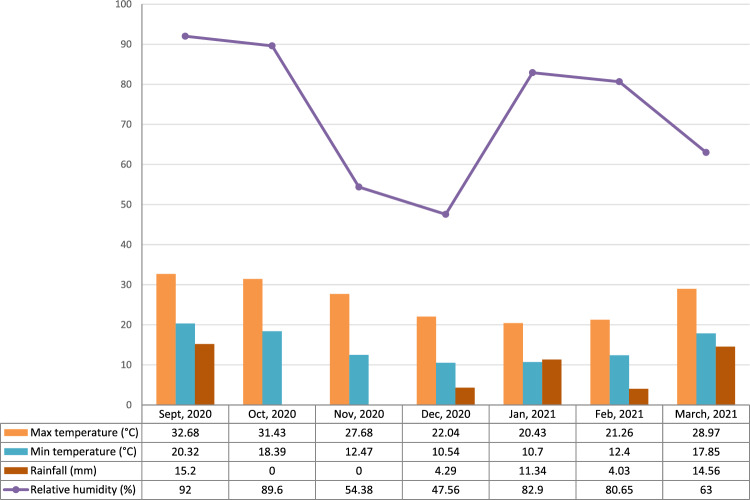
Meteorological data of winter season, 2020–2021.

**Figure 3:**
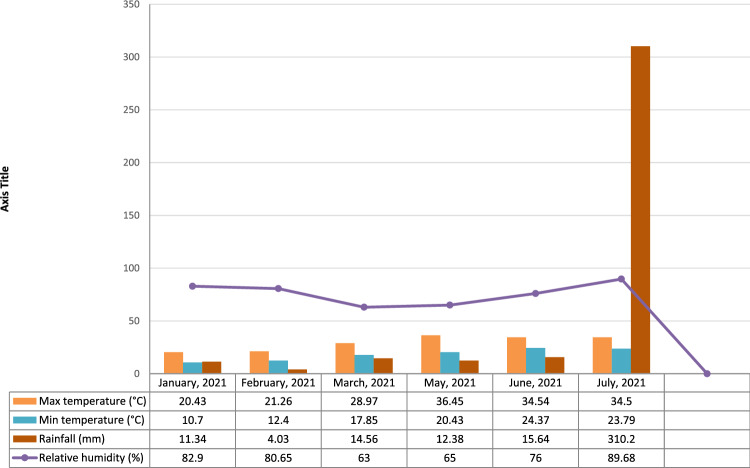
Meteorological data of summer season, 2021.

The investigation was carried out during summer and winter seasons of 2020 and the summer season of 2021 at RHRT&S of Dr YSP UHF, Dhaulakuan, Sirmour (HP), India. The investigation site is located at an elevation of 468 m amsl in agro-climatic Zone 1 of Himachal Pradesh. Meteorological data of experimental site for all three seasons have been presented in Figs. 1, 2, 3

### Experimental material

The Local chilli landraces (confined to the kitchen gardens) were collected from different villages of Sirmour district of Himachal Pradesh and compared with the recommended cultivar DKC-8. The landraces along with their sources of collection have been presented in Table 1.

**Table 1:** List of chilli landraces along with their sources of collection.

Genotype	Source
CS1	Kando (Shillai) Sirmour (HP)
CS2	Kuffar (Shillai) Sirmour (HP)
CS3	Dimti (Shillai) Sirmour (HP)
CS4	Bandli (Shillai) Sirmour (HP)
CS5	Forrar (Shillai) Sirmour(HP)
CS6	Dadhas (Shillai) Sirmour(HP)
CS7	Dudhog(Shillai) Sirmour(HP)
CS8	Aeraana (Shillai) Sirmour(HP)
CS9	Choila (Shillai) Sirmour(HP)
CS10	Patan(Shillai) Sirmour(HP)
CS11	Ghasan (Shillai), Sirmour (HP)
CS12	Bali(Shillai) Sirmour(HP)
CS13	Bhatnol(Shillai) Sirmour(HP)
CS14	Chakri (Shillai) Sirmour(HP)
CS15	Nera (Shillai), Sirmaur(HP)
CS16	Pandhog (Shillai) Sirmaur(HP)
CS17	Gitaddi (Shillai) Sirmaur(HP)
CS18	Gawali (Shillai) Sirmaur(HP)
CS19	Kandiyari (Shillai) Sirmour (HP)
DKC-8	Department of Vegetable Science (UHF NAUNI)

*CS* Collection from Sirmour.

### Observation

The observations referring to ripe fruit characteristics were cataloged from five selected plants per landrace per replication and their means were analyzed statistically. All the landraces were assessed for the following traits: Days to 50 per cent flowering, Days to ripe fruit maturity, Plant height, Number of ripe fruits plant^-1^, Average ripe fruit weight (g), Ripe fruit yield plant^−1^(g).

### Statistical analysis

The data recorded was analyzed by using MS-Excel and OPSTAT. The mean values of each genotype in each replication for all the traits under study were subjected to statistical analysis as per Randomized Complete Block Design.

#### Analysis of variance

The data collected on different characteristics was processed for the Analysis of Variance as suggested by Panse and Sukhatme^5^. The Table 2 for analysis of variance (ANOVA) was set as explained by Gomez and Gomez^6^.

**Table 2:** ANOVA for RCBD (Randomized Complete Block Design).

Source of variation	Degree of freedom	Sum of squares	Mean sum of squares	F (cal)
Replications (r)	(r−1)	S_r_	Sr/r−1 = ${{\text{M}}}_{{\text{r}}}$	${{\text{M}}}_{{\text{r}}}/{{\text{M}}}_{{\text{e}}}$
Genotypes (g)	(g−1)	S_g_	Sg/g−1 = ${{\text{M}}}_{{\text{g}}}$	${{\text{M}}}_{{\text{g}}}/{{\text{M}}}_{{\text{e}}}$
Error (e)	(r−1)(g−1)	S_e_	Se/(r−1) (g−1) = ${{\text{M}}}_{{\text{e}}}$	

where r = Number of replications, g = Number of genotypes, Sr = Sum of squares due to replications, Sg = Sum of squares due to genotypes, Se = Sum of squares due to error, Mr = Mean sum of squares due to replications, Mg = Mean sum of squares due to genotypes, Me = Mean sum of squares due to error.

The replications and entries mean sum of squares were tested against mean sum of squares due to error by F test with (r−1), (r−1) (g−1) and (g−1), (r−1) (g−1) degrees of freedom at 5 per cent level of significance.

The calculated F- values were compared with tabulated F- value. When F-test was found significant, critical difference was calculated to find out the superiority of one treatment over the others.

The standard errors and critical differences were calculated as follows:

$${\text{SE }}\left( {\text{m}} \right) \, \pm = \sqrt {{\text{Me}}/{\text{r}}}$$

$${\text{SE }}\left( {\text{d}} \right) \, \pm = \sqrt {2{\text{Me}}/{\text{r}}}$$

$${\text{CD}}_{{0.0{5}}} = {\text{S}}.{\text{E}}. \, \left( {\text{d}} \right) \times {\text{ t}}_{{(0.0{5})}} \left( {{\text{r}} - {1}} \right) \, \left( {{\text{g}} - {1}} \right){\text{ df}}$$

where $${\text{SE }}\left( {\text{m}} \right) \pm = {\text{Standard}}\,{\text{error}}\,{\text{of}}\,{\text{mean}}$$, $${\text{SE }}\left( {\text{d}} \right) \pm = {\text{Standard}}\,{\text{error}}\,{\text{of}}\,{\text{difference}}$$, $${\text{CD}}_{{0.0{5}}} = {\text{Critical}}\,{\text{difference}}\,{\text{at}}\,{5}\,{\text{per}}\,{\text{cent}}\,{\text{level}}\,{\text{of}}\,{\text{significance}}$$

#### Parameters of variability

Parameters of variability were estimated as per formula given by Burton and Devane^7^.

(A) Phenotypic coefficient of variation (PCV)
  $$\mathrm{PCV }(\mathrm{\%})=\frac{\sqrt{\mathrm{Phenotypic\, variance\, }({\text{Vp}})}}{\mathrm{General\, mean\, of\, population\, }({\text{GM}})}\mathrm{x }100$$
(B) Genotypic coefficient of variation (GCV)
  $$\mathrm{GCV }(\mathrm{\%}) =\frac{\sqrt{\mathrm{Genotypic\, variance\, }({\text{Vg}})}}{\mathrm{General\, mean\, of\, population\, }({\text{GM}})}\mathrm{x }100$$
  where V_e_ = M_e_, V_g_ = Genotypic variance (M_g_—M_e_)/r, V_p_ = Phenotypic variance (V_g_ + V_e_).

PCV and GCV were interpreted as shown below (Cherian 2000)

$${\text{Less}}\,{\text{than}}\,{15}\% = {\text{Low}}$$

$${15} - {3}0\% = {\text{Moderate}}$$

$${\text{More}}\,{\text{than}}\,{3}0\% = {\text{High}}$$

#### Heritability

Heritability in broad sense was calculated as per the formula given by Burton and Devane^7^.

$${\text{H}}\left( \% \right) = \frac{{{\text{Vg}}}}{{{\text{Vp}}}}$$

where H = Heritability, V_g_ = Genotypic variance (M_g_–M_e_)/r, V_p_ = Phenotypic variance (V_g_ + V_e_).

#### Genetic advance

The expected genetic advance resulting from selection of five per cent superior individuals was calculated:

$${\text{GA}} = {\text{H }} \times \sigma {\text{p}} \times {\text{K}}$$

where H = Heritability (%), σp = Phenotypic standard deviation, K = Selection differential at 5% selection index (K = 2.06).

#### Correlation analysis

The correlations between all characters under study, at genotypic, phenotypic and environmental level were estimated as per the method described by Al-Jibouri et al.^8^. The characters which were showing non-significant difference ANOVA were not taken for studying correlation (Table 3).

**Table 3:** ANOVA for Correlation coefficients.

Source of variation	Degree of freedom	Mean sum of square		Mean sum of square	Variation ratio (F—value)
		X	Y		
Replications (r)	(r−1)				
Genotypes (g)	(g−1)	M_g_ X	M_g_ Y	M_g_ XY = MP_1_	*MP*_1_/*MP*_2_
Error (e)	(r−1)(g−1)	M_e_ X	M_e_ Y	M_e_ XY = MP_2_	

Environmental covariance (V_e_XY) = MP_2_.

Genotypic covariance (V_g_XY) = (MP_1_–MP_2_)/r.

Phenotypic covariance (V_p_XY) = V_g_XY + V_e_XY.

where V_e_XY = Environmental covariance between X and Y, V_g_XY = Genotypic covariance between X and Y, V_p_XY = Phenotypic covariance between X and Y.

Coefficients of correlation

1. Phenotypic correlation between characters X and Y:
  $$\mathrm{rp }=\frac{VpXY}{\sqrt{VpX \times VpY}}$$
2. Genotypic correlation between characters X and Y:
  $${\text{rg}}=\frac{{\text{VgXY}}}{\sqrt{{\text{VgXxVgY}}}}$$
3. Environmental correlation between characters X and Y:
  $${\text{re}}=\frac{{\text{VeXY}}}{\sqrt{{\text{VeXxVeY}}}}$$

V_p_XY, V_g_XY and V_e_XY denotes phenotypic, genotypic and environmental covariances between characters X and Y, respectively.

V_p_X, V_g_X, V_e_X denotes phenotypic, genotypic and environmental covariances between characters X, whereas, V_p_Y, V_g_Y, V_e_Y denotes phenotypic, genotypic and environmental variances between characters Y.

#### Path coefficient analysis

The following formula was used for calculating path coefficient analysis suggested by Dewey and Lu^9^. The path coefficient was obtained by the simultaneous selection of following equations, which express the basic relationship between genotypic correction (r) and path coefficient (P)

$${\text{r}}_{{{14}}} = {\text{ P}}_{{{14}}} + {\text{ r}}_{{{12}}} {\text{P}}_{{{24}}} + {\text{ r}}_{{{13}}} {\text{P}}_{{{34}}}$$

$${\text{r}}_{{{24}}} = {\text{ r}}_{{{21}}} {\text{P}}_{{{14}}} + {\text{ P}}_{{{24}}} + {\text{ r}}_{{{23}}} {\text{P}}_{{{34}}}$$

$${\text{r}}_{{{34}}} = {\text{ r}}_{{{31}}} {\text{P}}_{{{14}}} + {\text{ P}}_{{{32}}} + {\text{ r}}_{{{24}}} {\text{P}}_{{{34}}}$$

where r_14_, r_24_ and r_34_ were genotypic correlation of components characters with yield (dependent variable) and r_13_, r_23_ and r_24_ were genotypic correlations among the component characters (independent variable) and r_12_ P_24_, r_13_ P_34_, r_21_ P_14_, r_31_ P_14_ and r_24_ P_34_ indirect effects.

The direct effects were calculated by the following set of equations:

$${\text{P}}_{{{14}}} = {\text{ C}}_{{{11}}} {\text{r}}_{{{14}}} + {\text{ C}}_{{{12}}} {\text{r}}_{{{24}}} + {\text{ C}}_{{{13}}} {\text{r}}_{{{34}}}$$

$${\text{P}}_{{{24}}} = {\text{ C}}_{{{31}}} {\text{r}}_{{{14}}} + {\text{ C}}_{{{32}}} {\text{r}}_{{{32}}} + {\text{ C}}_{{{23}}} {\text{r}}_{{{34}}}$$

$${\text{P}}_{{{34}}} = {\text{ C}}_{{{31}}} {\text{r}}_{{{14}}} + {\text{ C}}_{{{32}}} {\text{r}}_{{{32}}} + {\text{ C}}_{{{24}}} {\text{r}}_{{{34}}}$$

where C_11_, C_12_, C_23_ and C_33_ were constants and P_14_, P_24_ and P_34_ were the estimates of direct effects.

*Residual effect*: It measures the role of other possible independent variables which were not included in the study on dependent variable. The residual effect was estimated with the help of direct effect and simple correction coefficient as given below:

$${\text{I }} = {\text{ P}}^{{2}} {\text{x}}_{{4}} + {\text{ P}}^{{2}}_{{{14}}} + {\text{ P}}^{{2}}_{{{24}}} + {\text{ P}}^{{2}}_{{{34}}} + {\text{ 2P}}_{{{14}}} {\text{r}}_{{{12}}} {\text{P}}_{{{34}}} + {\text{ 2P}}_{{{24}}} {\text{r}}_{{{22}}} {\text{P}}_{{{34}}}$$

#### Stability analysis

The mean value recorded for different characters in respect of 20 genotypes in 3 environments as well pooled over the environments, were used for analysis of variance for phenotypic.

Analysis of variance for stability parameters were showed in Table 4.

**Table 4:** Analysis of variance for stability parameters.

Source of variation	Degree of freedom	Sum of squares	Mean sum of squares
Total	Et−1	∑∑ Y_ij_^2^–CF ij	
Genotypes	(t−1)	I/E ∑ Y_i_^2^–CF i	MS_1_
Environments	E−1	∑∑ Y_ij_^2^/E ij	
Env. + (Geo. X Env.)	t (E−1)	I/t (∑ Y_ij_)^2^/∑ I^2^_j_ jj	
Environment (linear)	1		
Geno. X Env. (linear)	(t−1)	∑[(∑ Y_ij_Ij)^2^/∑ I^2^_j_]—Env. (lin.) S.S. ij j	MS_2_
Pooled deviation	t (E−2)	∑∑$\delta$^2^_ij_ i j	MS_3_
Pooled error	E (t−1) (r−1)		MS_4_

t = Treatments (genotypes), E = Environments, MS_1_ = Treatments (genotypes) mean sum of squares, MS_2_ = G × E interaction mean sum of squares, MS_3_ = Pooled deviation mean sum of squares, MS_4_ = Pooled error mean sum of squares.

The methods of stability analysis used in this investigation were suggested by Eberhart and Russell^10^.

$${\text{Y}}_{{\text{i j}}} = \mu_{{\text{i}}} + \beta {\text{i Ij }} + \sigma_{{{\text{ij}}}}^{2}$$

where Y_ij_ = Mean of ith variety in jth environment (I = 1,2,… t) and (j = 1,2,…E), $\beta$_i_ = Regression coefficient that measure the response of the ith variety of the varying environment. I_j_ = Environment index of all the jth environment and is obtained as deviation of mean of all genotypes of the jth environment from over all mean. $\sigma$^2^_ij_ = Deviation from regression of the ith variety at the jth environment.

Stability parameters

The three parameters of stability are

(i) Mean
(ii) bi
(iii) S^2^ di

Regression coefficient (bi)

The regression coefficient (bi) is the regression at the performance of each variety under different environments on the environmental mean over all the genotypes.

$${\text{bi}} = \sum_{{\text{j}}} {\text{Y}}_{{{\text{ij}}}} {\text{I}}_{{\text{j}}} / \, \sum_{{\text{j}}} {\text{I}}_{{\text{j}}}^{2}$$

where ∑_j_ Y_ij_ I_j_ = Sum of products and $\sum_{{\text{j}}} {\text{I}}_{{\text{j}}}^{2}$ = Sum of squares of environmental index (I_j_).

Mean square deviation (S^2^ di) from linear regression

$${\text{S}}^{{2}} {\text{di }} = \sum_{{\text{j}}} \delta_{{{\text{ij}}}}^{2} /\left( {{\text{S}} - {2}} \right) - {\text{S}}^{{2}} {\text{e}}/{\text{r}}$$

where ∑_j_$\delta$^2^_ij_ = [∑_j_ Y^2^_ij_–Y_i_^2^/t]–(∑_j_ Y_ij_ I_j_)^2^/∑_j_ I_j_^2^, S^2^e** = **pooled error, r = number of replication, S = number of environments.

If these values are significantly deviating from zero, the expected cannot be predicted satisfactorily (unstable). When deviations are not significant, the conclusion may be drawn by considering jointly the mean yield and regression value^10^ as showen in Table 5:

**Table 5:** Conclusion for stability analysis.

Regression coefficient (bi)	Mean value of trait (ų)	Stability	Remarks
b = 1	High	Average	Well adapted to all environments
b = 1	Low	Average	Poorly adapted to all environments
b > 1	High	Below average	Specifically adapted to favourable environments
b < 1	High	Below average	Specifically adapted to unfavourable environments

According to Eberhart and Russell^10^, the highly stable hybrid is one with high mean above the population mean (p), regression coefficient (b) = 1.00 and non significant deviation from regression (S2d) = 0 The estimate of deviation from regression (using F test) suggests the degree of reliance that should be put on linear regression in interpretation of the data.

**Figure 4:**
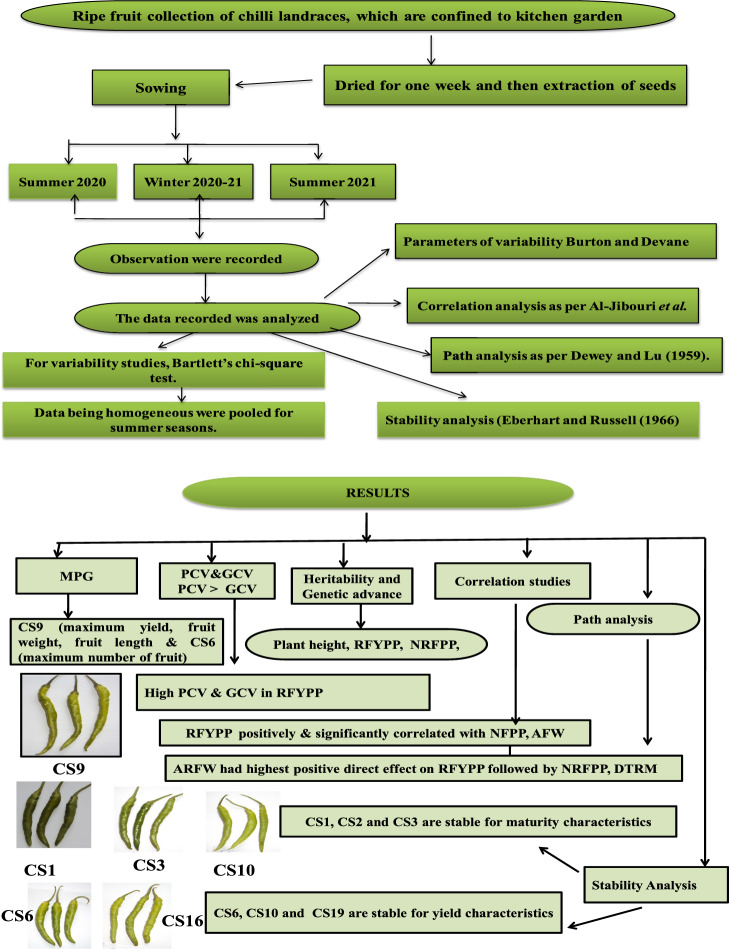
Graphical architecture of the research.

Figure 4 showed the Graphical Architecture of the Research.

## Results and discussions

### Variability studies

Variability study for different characteristics of chilli landraces was done by Bartlett’s chi square test for testing the goodness of fit and test of homogeneity. Since Bartlett’s test is insignificant for summer seasons 2020 and 2021, therefore it was pooled for these two seasons. Data were heterogeneous when Bartlett’s test was implied on three seasons, hence the inferences for winter season are drawn separately.

#### Mean performance of the genotypes

The analysis of variance indicated significant variations among genotypes for all the characteristics studied which showed that the material contains considerable genetic variability. The mean performance of 20 genotypes for the various characteristics has been described and discussed below:

##### Days to 50 per cent flowering:

In summer seasons (Table 6), days to 50 per cent flowering ranged from 41.00–61.50 days with the population mean 53.03** ± **0.87 days. Genotypes CS7 and CS9 (41.00 days) were earliest in flowering followed by CS13 (44.50 days). Genotype CS15 (44.83 days) was also in close proximity. Maximum days to 50 per cent flowering were observed in genotype CS8 (61.50 days) and were statistically at par with CS5 (61.00 days) and CS17 (59.33 days). In winter season, days taken to 50 per cent flowering ranged from 75.00 to 97.67 days with the population mean 83.45** ± **0.76 days. Genotype CS3 (75.00 days) was earliest in flowering and in close proximity with CS15 (76.00 days), CS13 (76.33 days), CS7 (76.67 days) and CS9 (77.33 days). Maximum days have been taken to 50 per cent flowering by the genotype CS8 (97.67 days). In winter season, genotypes took more days to flowering. It might be due to low temperature during December to mid-January and hence during this period plants growth remained dormant. After January, with the rising temperature flowering initiated in the given landraces. In both the seasons, different genotypes exhibited differential performance due to the environmental effects of summer and winter.

**Table 6:** Mean performance of chilli genotypes for days to 50 per cent flowering in summer and winter seasons.

Seasons	Seasons			
	Summer 2020	Summer 2021	Pooled mean	Winter 2020
CS1	51.33	51.00	51.17	79.00
CS2	56.33	56.67	56.50	80.67
CS3	46.33	47.67	47.00	75.00
CS4	56.00	55.67	55.83	85.33
CS5	61.33	60.67	61.00	90.67
CS6	52.00	52.33	52.17	83.33
CS7	40.67	41.33	41.00	76.67
CS8	62.00	61.00	61.50	97.67
CS9	41.00	41.00	41.00	77.33
CS10	50.00	51.00	50.50	81.67
CS11	59.00	58.33	58.67	93.33
CS12	55.33	55.67	55.50	86.67
CS13	45.00	44.00	44.50	76.33
CS14	55.67	56.67	56.17	80.67
CS15	45.00	44.67	44.83	76.00
CS16	54.33	55.67	55.00	80.00
CS17	60.00	58.67	59.33	92.33
CS18	56.00	53.33	54.67	83.67
CS19	57.00	55.33	56.17	85.00
DKC-8	57.67	58.33	58.00	87.67
Range	40.67–62.00	41.00–61.00	41.00–61.50	75.00–97.67
Mean	53.10	52.95	53.03	83.45
± SE(m)	1.15	0.92	0.87	0.76
C.D _(P=0.05)_	3.31	2.63	2.49	2.19
Genotypes			2.75	
Seasons			NS	
Genotype × Seasons			NS	

*CS* Collection from Sirmour, *NS* non-significant, *CD* critical difference, *SE(m)* Standard Error due to mean.

##### Days to ripe maturity

In summer seasons (Table 7), significant variations were observed among all the genotypes for this characteristic which ranged from 90.00 to 116.50 days and the population mean was 104.78** ± **1.19 days. Genotype CS13 (90.00 days) was the earliest to produce red fruits and was close proximity with CS7 (91.17 days). Genotype CS8 (116.50 days) took maximum days to produce ripe fruits, which was statistically at par with CS17 (115.33 days) and CS16 (113.33 days).In winter season, days to ripe maturity ranged from 128.67 to 143.33 days with the population mean 135.63** ± **0.62 days. Genotype CS14 (128.67 days) was earliest to produce red ripe fruits and was statistically at par with CS3 (129.33 days) and CS10 (130.33 days). Genotype CS8 (143.33 days) took maximum days to produce red ripe fruits. In summer seasons, CS13 took 44.50 days for 50 per cent flowering and 68.50 days to green maturity. In winter season, CS14 took 80.67 days for 50 per cent flowering and 102.00 days to green maturity. Ample variability with respect to this characteristic has been reported by various workers where the ripe maturity ranged from DKC-8 matured in 110 days after transplanting^11^ and 121.67–140.26 days^12^.

**Table 7:** Mean performance of chilli genotypes for days to maturity (mature ripe stage) in summer and winter seasons:

Genotypes	Seasons			
	Summer 2020	Summer 2021	Pooled mean	Winter 2020
CS1	99.67	98.67	99.17	134.33
CS2	102.00	102.67	102.33	135.67
CS3	96.33	94.67	95.50	129.33
CS4	107.00	108.33	107.67	139.33
CS5	113.67	111.33	112.50	136.33
CS6	102.33	103.33	102.83	137.67
CS7	92.00	90.33	91.17	132.33
CS8	117.33	115.67	116.50	143.33
CS9	99.67	99.00	99.33	134.67
CS10	104.33	103.67	104.00	130.33
CS11	112.67	111.33	112.00	137.33
CS12	104.00	103.00	103.50	140.00
CS13	90.67	89.33	90.00	138.00
CS14	107.33	108.67	108.00	128.67
CS15	97.33	97.67	97.50	133.33
CS16	113.00	113.67	113.33	131.33
CS17	114.33	116.33	115.33	141.33
CS18	110.67	109.67	110.17	135.33
CS19	108.67	109.33	109.00	138.33
DKC-8	106.33	105.33	105.83	135.67
Range	90.67–117.33	89.33–115.67	90.00–116.50	128.67–143.33
Mean	104.97	104.60	104.78	135.63
± SE(m)	1.71	0.93	1.19	0.62
C.D _(*P* = 0.05)_	4.90	2.68	3.43	1.79
Genotypes			3.63	
Seasons			NS	
Genotype × Seasons			NS	

*CS* collection from Sirmour, *NS* non-significant, *CD* critical difference, *SE(m)* standard error due to mean.

##### Plant height (cm)

In summer seasons (Table 8), plant height of the genotypes ranged from 63.92–102.71 cm and the population mean was 81.42** ± **0.45 cm. Genotype CS10 showed maximum plant height (102.65 cm) and had a non-significant difference with CS15 (97.99 cm). Minimum plant height was observed in genotype CS3 (63.92 cm) which was statistically at par with DKC-8 (64.94 cm) and CS16 (65.16 cm). In winter season, plant height of the genotypes ranged from 57.31 to 97.20 cm and the population mean was 75.68** ± **1.03 cm. Genotype CS10 showed maximum height of plant (97.20 cm) followed by CS8 (93.11 cm). Genotype CS15 (92.26 days) was also in close proximity. Minimum plant height was observed in genotype CS16 (57.31 cm) which was statistically at par with CS3 (58.33 cm) and DKC-8 (59.35 cm).Plant height was recorded maximum in summer season than that of winter season.

**Table 8:** Mean performance of chilli genotypes for plant height (cm) in summer and winter season.

Genotypes	Seasons			
	Summer 2020	Summer 2021	Pooled mean	Winter 2020
CS1	94.43	93.94	94.18	86.69
CS2	83.22	82.67	82.95	77.53
CS3	64.06	63.77	63.92	58.33
CS4	73.53	73.21	73.37	67.65
CS5	78.49	78.00	78.24	77.54
CS6	91.27	91.21	91.24	85.85
CS7	84.52	84.51	84.52	80.67
CS8	93.79	94.76	94.27	93.11
CS9	68.89	67.94	68.42	62.46
CS10	102.79	102.62	102.71	97.20
CS11	93.22	92.61	92.92	86.28
CS12	71.51	70.39	70.95	67.03
CS13	80.72	80.34	80.53	73.39
CS14	70.94	71.25	71.10	62.08
CS15	98.54	97.44	97.99	92.26
CS16	64.87	65.45	65.16	57.31
CS17	84.17	83.91	84.04	77.97
CS18	76.24	75.95	76.10	66.23
CS19	90.41	91.45	90.93	84.72
DKC-8	65.43	64.46	64.94	59.35
Range	64.06–102.79	63.77–102.62	63.92–102.71	57.31–97.20
Mean	81.55	81.29	81.42	75.68
± SE(m)	0.45	0.61	0.45	1.03
C.D _(*P* = 0.05)_	1.31	1.74	1.29	2.95
Genotypes			1.41	
Seasons			NS	
Genotype × Seasons			NS	

*CS* collection from Sirmour, *NS* non-significant, *CD* critical difference, *SE(m)* standard error due to mean.

In winter season, plant remained dormant due to which photosynthesis and respiration rate decreased due to unfavorable climate resulting into minimum plant height. Further, under stress, plants entered reproductive phase earlier as caused by low temperature in winter. Wide range of variability with respect to plant height has been reported by various workers where plant height ranged from 73.70 to 99.30 cm^13^, 54.80–119 cm^14^.

##### Fruiting habit

Two types of fruit habit were observed in the genotypes viz., drooping and upright has been presented in Table 9. It was observed that out of twenty genotypes, eighteen genotypes showed drooping fruiting and only two genotypes showed upright fruiting. These results are similar with the findings of Srivastava et al.^15^ and Joshi et al*.*^16^ varying from drooping to upright fruiting habit.

**Table 9:** Mean performance of chilli genotypes for fruiting habit and fruit blossom end shape.

Genotypes	Fruiting habit	Fruit blossom end shape
CS1	Drooping	Pointed
CS2	Drooping	Pointed
CS3	Upright	Pointed
CS4	Drooping	Pointed
CS5	Drooping	Pointed
CS6	Drooping	Pointed
CS7	Drooping	Pointed
CS8	Drooping	Pointed
CS9	Drooping	Pointed
CS10	Drooping	Pointed
CS11	Drooping	Pointed
CS12	Drooping	Pointed
CS13	Drooping	Pointed
CS14	Drooping	Pointed
CS15	Drooping	Pointed
CS16	Drooping	Pointed
CS17	Drooping	Pointed
CS18	Drooping	Pointed
CS19	Drooping	Pointed
DKC-8	Upright	Pointed

*CS* collection from Sirmour.

##### Fruit blossom end shape

The data in Table 9 showed that all the genotypes showed pointed fruit blossom end shape. Orobiyi et al.^17^ and Srivastava et al.^15^ had also recorded similar blossom end shape in chilli fruits.

##### Number of ripe fruits plant^−1^

In summer seasons, number of ripe fruits plant^−1^ varied significantly in all the genotypes from 32.00 to 87.13 and the population mean was 64.10 ± 1.31 as presented in Table 10. Maximum number of ripe fruits plant^−1^ was observed in genotype CS6 (87.13) which was statistically at par with CS18 (85.89) and CS13 (85.19). Minimum number of ripe fruits per plant was observed in genotype CS5 (32.00) followed by CS8 (40.69). Number of ripe fruits plant^−1^ in winter season varied from 27.00 to 82.27 and the population mean was57.30 ± 0.60. Maximum number of ripe fruits per plant was observed in genotype CS6 (82.67) and was in close proximity with CS18 (79.70), CS13 (78.40). Minimum number of ripe fruits plant^-1^was observed in genotype CS5 (27.00). Ample variability with respect to this parameter has been reported by various workers where number of ripe fruits per plant ranged from 38.46 to 223.16^18^ and 90.50–214.20^14^.

**Table 10:** Mean performance of chilli genotypes for number of ripe fruits plant^−1^.

Genotype	Seasons			
	Summer 2020	Summer 2021	Pooled mean	Winter 2020
CS1	55.60	53.17	54.39	50.53
CS2	61.19	63.01	62.10	56.70
CS3	77.28	77.88	77.58	71.27
CS4	49.07	51.10	50.09	44.13
CS5	32.23	31.78	32.00	27.00
CS6	88.92	85.34	87.13	82.27
CS7	65.22	63.94	64.58	60.93
CS8	41.45	39.93	40.69	32.87
CS9	66.44	67.99	67.22	59.07
CS10	66.45	65.19	65.82	62.66
CS11	61.20	63.68	62.44	53.80
CS12	51.35	52.37	51.86	40.22
CS13	84.65	85.73	85.19	78.40
CS14	64.67	64.63	64.65	59.60
CS15	64.02	63.24	63.63	58.07
CS16	66.23	67.40	66.82	55.67
CS17	46.98	49.21	48.10	39.53
CS18	85.68	86.10	85.89	79.70
CS19	77.34	80.02	78.68	65.23
DKC-8	72.48	73.73	73.10	68.39
Range	32.23–88.92	31.78–85.34	32.00–87.13	27.00–82.27
Mean	63.92	64.27	64.10	57.30
± SE(m)	1.24	1.84	1.31	0.60
C.D_(*P* = 0.05)_	3.55	5.30	3.78	1.70
Genotypes			4.15	
Seasons			NS	
Genotype × Seasons			NS	

*CS* collection from Sirmour, *NS* non-significant, *CD* critical difference, *SE(m)* standard error due to mean.

##### Average ripe fruit weight (g)

Data obtained on average ripe fruit weight for pooled mean in summer seasons revealed the presence of significant variation among the genotypes ranged from 1.95 to 5.01 with the population mean 3.18 ± 0.07 as presented in Table 11. Maximum average ripe fruit weight was recorded in genotype CS9 (5.01 g) followed by CS15 (4.77 g). Genotype CS8 (4.70 g) was also in close proximity. Minimum value was observed in genotype CS7 (1.95 g) which was statistically at par with CS1 (2.01 g) and CS2 (2.12 g).

**Table 11:** Mean performance of chilli genotypes for average ripe fruit weight (g) plant^−1^.

Genotype	Seasons			
	Summer 2020	Summer 2021	Pooled mean	Winter 2020
CS1	1.99	2.03	2.01	1.97
CS2	2.10	2.14	2.12	2.09
CS3	2.45	2.44	2.45	2.30
CS4	2.70	2.68	2.69	2.53
CS5	3.67	3.71	3.69	3.34
CS6	2.70	2.74	2.72	2.66
CS7	1.95	1.94	1.95	1.92
CS8	4.69	4.70	4.70	4.58
CS9	5.01	5.02	5.01	5.02
CS10	3.93	3.89	3.91	3.72
CS11	2.93	2.87	2.90	2.89
CS12	3.41	3.45	3.43	3.23
CS13	3.12	3.15	3.14	2.86
CS14	2.77	2.80	2.79	2.76
CS15	4.76	4.78	4.77	4.63
CS16	3.70	3.72	3.71	3.58
CS17	2.73	2.78	2.75	2.66
CS18	3.34	3.34	3.34	3.35
CS19	2.79	2.77	2.78	2.73
DKC-8	2.74	2.75	2.75	2.72
Range	1.95–5.01	1.94–5.02	1.95–5.01	1.92–5.02
Mean	3.17	3.18	3.18	3.08
± SE(m)	0.07	0.06	0.07	0.074
C.D _(*P* = 0.05)_	0.21	0.22	0.21	0.12
Genotypes			0.19	
Seasons			NS	
Genotype × Seasons			NS	

*CS* collection from Sirmour, *NS* non-significant, *CD* critical difference, *SE(m)* standard error due to mean.

In winter season, average ripe fruit weight of all the genotypes varied from 1.95 to 5.02 with the population mean 3.08 ± 0.074. Maximum average ripe fruit weight was recorded in genotype CS9 (5.02 g) followed by CS15 (4.63 g). Genotype CS8 (4.58 g) was in closeproximity with CS15. The minimum value was observed in genotype CS7 (1.92 g) which was statistically at par with CS1 (1.97 g). Ample variability with respect to this parameter has been reported by Dhaliwal et al.^19^ had recorded a range from 1.5 to 4.00 g, Sahu et al.^20^ from 3.43 to 8.97 g and Negi and Sharma^21^ from 21.11 to 5.64 g.

##### Ripe fruit yield plant^−1^ (g)

In summer seasons, ripe fruit yield varied from 109.20 to 336.88 g and the population mean of 201.33 ± 5.22 g as presented in Table 12. Genotype CS9 produced maximum ripe fruit yield per plant (336.88 g) followed by CS15 (303.74 g) and CS18 (286.62 g). Minimum ripe fruit yield was observed in genotype CS1 (109.20 g) and was statistically at par with CS5 having ripe fruit yield per plant 117.83 g. Whereas in winter season, ripe fruit yield varied from 90.23 to 296.30 g and the population mean was 173.87 ± 3.29 g. Genotype CS9 produced maximum ripe fruit yield per plant (296.30 g) followed by CS15 (268.58 g) and CS18 (266.73 g). Minimum ripe fruit yield was observed in genotype CS5 (90.23 g) which was statistically at par with CS1 having ripe fruit yield per plant 99.38 g. Genotype CS9 produced maximum ripe fruit yield in in summer and winter seasons due to maximum fruit length, fruit girth and higher average fruit weight. Further, variation in yield of summer and winter was due to less number of fruits reaped in winter season. Other workers recorded higher in ripe fruit yield viz. Dhaliwal et al.^19^ reported 145.00–586.00 g, Joshi and Nabi^22^ reported 630.50–1057 g and Negi and Sharma^21^ reported 48.31–168.83 g.

**Table 12:** Mean performance of chilli genotypes for ripe fruit yield per plant (g) in summer and winter seasons:

Genotype	Seasons			
	Summer 2020	Summer 2021	Pooled mean	Winter 2020
CS1	110.63	107.76	109.20	99.38
CS2	128.27	134.79	131.53	118.31
CS3	189.41	189.55	189.48	164.16
CS4	132.52	136.58	134.55	111.84
CS5	118.00	117.67	117.83	90.23
CS6	240.09	234.13	237.11	219.14
CS7	127.42	124.40	125.91	116.80
CS8	194.35	187.58	190.96	150.59
CS9	332.65	341.11	336.88	296.30
CS10	261.19	253.66	257.42	232.87
CS11	179.22	182.75	180.98	155.48
CS12	174.96	180.10	177.53	129.77
CS13	264.04	270.29	267.16	223.96
CS14	179.48	180.85	180.16	164.71
CS15	304.93	302.55	303.74	268.58
CS16	245.30	250.25	247.77	199.25
CS17	128.16	136.34	132.25	105.07
CS18	285.86	287.37	286.62	266.73
CS19	215.78	221.49	218.64	178.23
DKC-8	198.56	203.03	200.79	186.02
Range	110.63–332.65	107.76–341.11	109.20–336.88	90.23–296.30
Mean	200.54	202.11	201.33	173.87
± SE(m)	5.95	6.15	5.22	3.29
C.D _(*P* = 0.05)_	17.11	17.67	15.02	9.45
Genotypes			15.99	
Seasons			NS	
Genotype × Seasons			NS	

*CS* collection from Sirmour, *NS* non-significant, *CD* critical difference, *SE(m)* standard error due to mean.

#### Phenotypic and genotypic coefficients of variation

**Figure 5:**
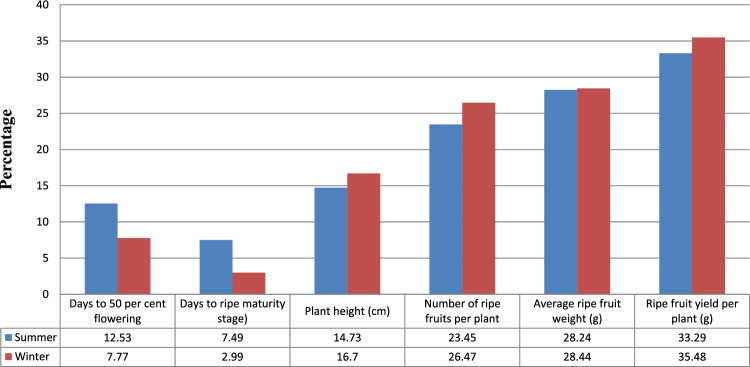
Graphical representation of Phenotypic coefficient of variation for summer and winter seasons.

**Figure 6:**
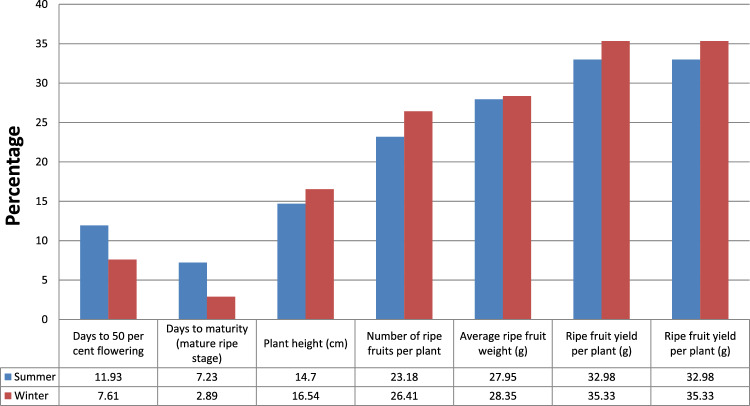
Graphical representation of Genotypic coefficient of variation for summer and winter seasons.

The values of Phenotypic coefficients of variation(PCV) were higher as compared to Genotypic coefficient of variation(GCV) but in close proximity as depicted in Table 13 and Fig. 5 (PCV) and Fig. 6 (GCV) which showed that the environmental effects were prevalent, but had meager effects on the appearance of the traits. High values of PCV and GCV (i.e. > 30%) were recorded for ripe fruit yield plant^−1^ (33.29, 32.98) and Values of phenotypic and genotypic coefficients of variation were moderate for ripe fruit weight (29.73, 29.54) and number of ripe fruits plant^−1^ (23.45, 23.18) during summer seasons. Whereas in winter season, phenotypic and genotypic coefficients of variationwere recorded maximum for ripe fruit yield plant^−1^ (35.48, 35.33) and also moderate for ripe fruit weight (28.44, 28.33) and number of ripe fruits plant^−1^ (26.47, 26.41). Ripe fruit yield in either of the seasons were greatly influenced by the seasonal effects. A significant variation was also observed among population mean of summer and winter seasons for days to 50 per cent flowering (53.03, 83.45), days to green maturity (78.83, 110.45) and days to ripe maturity (104.78, 135.63) respectively which exhibited the significant effect of environment for these characteristics. Low PCV and GCV (i.e. < 15%) in summer seasons were observed for plant height (14.70 and 14.73%), days to 50 per cent flowering (11.93 and 12.53%). and days to ripe maturity (7.23–7.49%). Whereas, low PCV and GCV in winter season were reported for days to 50 per cent flowering (7.61 and 7.77) and days to maturity (mature ripe stage) (2.89 and 2.99%). Low PCV and GCV indicated that genotypes possessed comparatively low genetic variation for these characteristics. Hence, these characteristics cannot be used for selection programmes.

**Table 13:** Estimation of phenotypic and genotypic coefficients of variation, heritability and genetic advance for various traits in chilli.

Characters	Range		PCV		GCV		Heritability (%)		Genetic advance	
	Summer	Winter	Summer	Winter	Summer	Winter	Summer	Winter	Summer	Winter
Days to 50 per cent flowering	41.00–61.50	75.00–97.67	12.53	7.77	11.93	7.61	90.63	95.84	12.32	12.80
Days to ripe maturity	90.00–116.50	128.67–143.33	7.49	2.99	7.23	2.89	93.05	92.92	15.05	7.78
Plant height (cm)	63.92–102.71	57.31–97.20	14.73	16.70	14.70	16.54	99.58	98.02	24.60	25.53
Number of fruits plant^−1^	32.00–87.13	27.00–82.87	23.45	26.47	23.18	26.41	97.71	99.54	30.26	31.11
Average fruit weight (g)	1.95–5.01	1.92–5.02	28.24	28.44	27.95	28.35	97.98	99.37	1.81	1.79
Fruit yield plant^−1^ (g)	109.20–336.88	90.23–296.30	33.29	35.48	32.98	35.33	98.18	99.15	135.53	126.01

*PCV* Phenotypic coefficient of variation, *GCV* genotypic coefficient of variation.

#### Heritability and genetic advance

**Figure 7:**
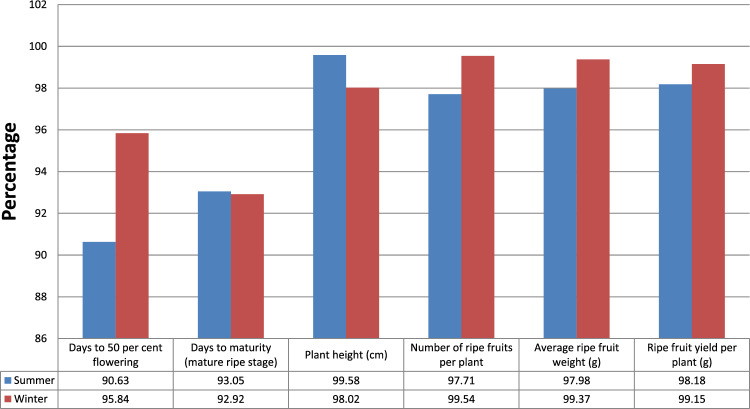
Graphical representation of heritability for summer and winter seasons.

**Figure 8:**
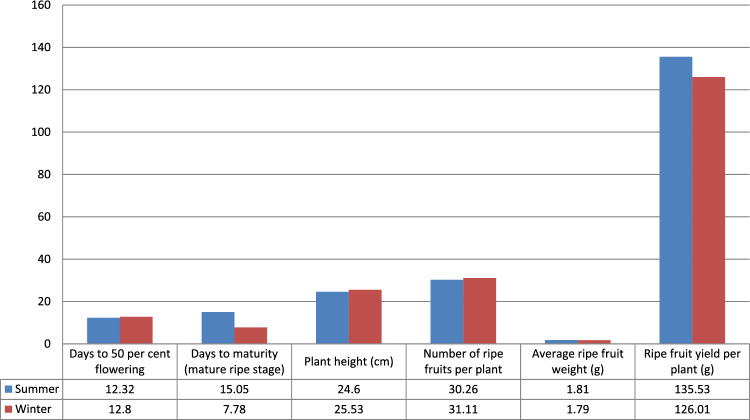
Graphical representation of Genetic advance for summer and winter seasons.

Data in Table 13, Figs. 7 and 8 are depicting high proportions of heritability (in broad sense) and genetic advance. The values were greater for ripe fruit yield per plant (98.18, 135.53) and number of ripe fruits (97.71, 30.26) in summer seasons. Whereas in winter season, heritability and genetic advance were recorded maximum for number of ripe fruits (99.54, 31.11) and ripe fruit yield plant^−1^ (99.15, 126.01) It is also contingent that summer and winter season could be exploited in assessing landraces, which will quicken the process of traditional breeding programme. But, obviously, plant height in summers and number of ripe fruits in winter will be the characteristics of focus. Selection on the basis of these characteristics could be effective for improving yield and these characteristics were controlled by additive genes. Results of high heritability and genetic advance were in accordance with earlier workers i.e. Nahak et al.^23^ reported high heritability and genetic advance for number of fruits per plant (93.43, 76.85) and fruit yield per plant (87.21, 57.90); Jyothi et al.^13^ reported for number of fruits per plant (97.45, 127.36) and ripe fruit yield plant^−1^ (94.14, 174.09).

### Correlation studies

Data in Tables 14 and 15 extrapolated that red ripe fruit yield plant^−1^ was positively and significantly correlated with number of fruits plant^−1^ and average fruit weight and significant and negative correlation with days to 50 per cent flowering. Whereas, in winter season, days to ripe maturity showed significant and negative correlation with ripe fruit yield plant^−1^. The findings are in accordance with the inferences made by Bijalwan and Mishra^24^.

**Table 14:** Genotypic and Phenotypic coefficients of correlation among different parameters in red chilli landraces in summer seasons.

Characters		DTFPF	DTMR	PH	NORF	ARFW
DTRM	G	0.827*				
	P	0.757*				
PH	G	0.080	0.013			
	P	0.078	0.011			
NORF	G	− 0.471*	− 0.450*	− 0.107		
	P	− 0.439*	− 0.424*	− 0.105		
ARFW	G	− 0.126	0.215	0.094	− 0.188	
	P	− 0.126	0.204	0.092	− 0.191	
RFYPP	G	− 0.497*	− 0.178	− 0.013	0.585*	0.675*
	P	− 0.468*	− 0.166	− 0.013	0.584*	0.672*

DTFPF, Days to 50% flowering; DTRM, Daysto ripe maturity; PH, Plant Height; NORF, Number of Ripe fruits Plant^−1^; ARFW, Average ripe fruit weight; RFYPP, Ripe fruit yield plant^−1^.

*Significant at 5 per cent level of significance; G, Genotypic level; P, Phenotypic level.

**Table 15:** Genotypic and Phenotypic coefficients of correlation among various characters in red chilli over winter season.

Characters		DTFPF	DTMR	PH	NORF	ARFW
DTMR	G	0.715*				
	P	0.679*				
PH	G	0.251	0.272*			
	P	0.247	0.262*			
NORF	G	− 0.583*	− 0.407*	− 0.171		
	P	− 0.571*	− 0.387*	− 0.171		
ARFW	G	0.129	0.102	0.125	− 0.192	
	P	0.125	0.098	0.122	− 0.190	
RFYPP	G	− 0.395*	− 0.272*	− 0.060	0.629*	0.628*
	P	− 0.388*	− 0.257*	− 0.061	0.629*	0.629*

DTFPF, Days to 50% flowering; DTRM, Days to ripe maturity; PH, Plant Height; NORF, Number of Ripe fruits Plant^−1^; ARFW, Average ripe fruit weight; RFYPP, Ripe fruit yield plant^−1^.

*Significant at 5 per cent level of significance; G, Genotypic level; P, Phenotypic level.

Number of ripe fruits per plant in summer and winter seasons showed negative and significant correlation with days to 50 per cent flowering and days to ripe maturity at genotypic and phenotypic level. Further, days to ripe maturity in summer and winter seasons showed positive and significant correlation with days to 50 per cent flowering at genotypic and phenotypic level. Plant height for winter season showed positive and significant correlation with days to ripe maturity.

The computation of correlation coefficients revealed that average ripe fruit weight and number of ripe fruits per plant were positively and significantly contributing characters to ripe fruit yield per plant. Although days to 50% flowering also played significant role in contributing to fruit yield but the direction was negative. It indicated that the early maturing genotypes (as observed in CS9 and CS7) could prove better yielders, because the earlier flowering enhanced fruiting duration.

### Path coefficient analysis

In summer seasons, path coefficients analysis in Table 16 extrapolated that average ripe fruit weight (ARFW) had the highest positive direct effects on ripe fruit yield plant^−1^ (RFYPP) followed by number of ripe fruits plant^−1^(NORF), days to ripe maturity (DRM) and plant height (PH), while negative direct effects on ripe fruit yield were observed by days to 50 per cent flowering. Whereas, highest positive indirect effects at genotypic level in summer seasons were observed on average fruit weight via days to ripe maturity, while the highest negative indirect effects recorded for number of ripe fruits via days to 50 per cent flowering and days to ripe maturity. Whereas in winter season (Table 17), average ripe fruit weight had the highest positive direct effects on ripe fruit yield followed by number of ripe fruits, while the highest negative direct effect on ripe fruit yield per plant was observed by plant height followed by days to 50 per cent flowering and days to ripe maturity. Whereas, the highest positive indirect effects at genotypic level were observed on average fruit weight via fruit girth and fruit length followed by fruit length via fruit girth. Maximum negative indirect effects were observed at number of fruits per plant via days to 50 per cent flowering, days to green maturity andfruit length. Positive direct effects of plant height on ripe fruit yield per plant had also been observed by Negi and Sharma^21^; negative direct effects of days to mature (red ripe stage) were recorded by Hasanuzzaman and Golam^25^ and negative direct effects of days to 50 per cent flowering on fruit yield were reported by Chattopadhyay et al.^26^.

**Table 16:** Direct and indirect effects of various parameters on yield of red chilli conducted over summer seasons.

Characters	DTFPF	DTMR	PH	NORF	ARFW	Genotypic correlation (r_g_) with RFYPP
DTFPF	− 0.151	0.081	0.000	− 0.330	− 0.097	− 0.49*
DTMR	− 0.125	0.098	0.000	− 0.316	0.165	− 0.17
PH	− 0.012	0.001	0.0003	− 0.075	0.072	− 0.01
NORF	0.071	− 0.044	− 0.000	0.702	− 0.144	0.58*
ARFW	0.019	0.021	0.000	− 0.132	0.766	0.67*

Residual effect = 0.02073; r_g_ = Genotypic correlation coefficient; Diagonal bold values are direct effects.

*Significant at 5 per cent level of significanceDTFPF, Days to 50% flowering; DTRM, Days to ripe maturity; PH, Plant Height; NORF, Number of Ripe fruits Plant^−1^; ARFW, Average ripe fruit weight; RFYPP, Ripe fruit yield plant^−1^.

**Table 17:** Direct and indirect effects of various prameters on yield of red chilli in winter season.

Characters	DTFPF	DTRM	PH	NORF	ARFW	Genotypic correlation (r_g_) with RFYPP
DTFPF	− 0.055	− 0.005	− 0.004	− 0.432	0.100	− 0.39*
DTRM	− 0.039	− 0.008	− 0.004	− 0.301	0.080	− 0.27*
PH	− 0.014	− 0.002	− 0.146	− 0.127	0.097	− 0.06
NORF	0.032	0.003	0.002	0.741	− 0.150	0.62*
ARFW	− 0.007	− 0.0008	− 0.002	− 0.142	0.780	0.62*

Residual effect = 0.02073; r_g_ = Genotypic correlation coefficient; Diagonal bold values are direct effects.

*Significant at 5 per cent level of significance; DTFPF, Days to 50% flowering; DTRM, Days to ripe maturity; PH, Plant Height; NORF, Number of Ripe fruits Plant^−1^; ARFW, Average ripe fruit weight; RFYPP, Ripe fruit yield plant^−1^.

### Genotype × environment interaction

A landrace does not exhibit the same phenotypic traits under different environments. G × E interaction isimportant for breeders in developing stable hybrids. Plant breeders are primarily concerned in improving productivity by multiplying the crop performance variations. Stability becomes quintessential in this situation^10^.

#### ANOVA for genotype × environment interaction

The pooled data over environments were analysed to estimate the interaction effects between genotypes × environment. The mean sum of squares for phenotypic stability for various characteristics has been shown in Table 18. The mean sum of squares due to genotypes, environments and genotypes × environment interaction were significant for all the characteristics. Environment (linear) mean sum of squares were significant for all the characteristics when tested against pooled deviation. Genotypes × environment (linear) interactions were also significant for all parameters except plant height.

**Table 18:** Pooled analysis of variance for stability of characters across seasons.

Source of variation	df	Days to 50% flowering	Days to ripe maturity	Plant height (cm)	Number of ripe fruits plant^−1^	Average ripe fruit weight (g)	Ripe fruit yield per plant (g)
Genotypes	19	112.20*	99.13*	441.31*	669.29*	2.35*	12,550.53*
Environment	2	6171.32*	6,345.49*	220.00*	308.52*	0.07*	5037.84*
Geno. × Environ	38	4.61*	17.44*	1.71*	3.32*	0.003*	59.02*
Environment (Linear)	1	12,342.63*	12,690.98*	440.00*	617.04*	0.14*	10,075.68*
Env. × Geno. (Linear)	19	8.67*	34.12*	3.22	5.29*	0.006*	106.57*
Pooled deviation	20	0.53	0.72	0.18	1.28	0.000	10.89
Pooled Error	114	2.74	4.12	1.63	5.27	0.013	0.39

*Significant at 5 per cent level of significance; df, degree of freedom.

#### Stability parameters

The stability parameters i.e. mean (x), regression coefficient (bi) and deviation from linear regression (S^2^di) for 20 landraces were worked out for horticultural characteristics to assess the stability of landraces over different seasons.

##### Days to 50 per cent flowering

The data in Table 19 showed that deviation from linear regression (S^2^di) was non-significant for all the genotypes indicating less contribution of linear regression toward G × E interaction. Genotypes CS7, CS9, CS13, CS15, CS3, CS1, CS10 and CS6 had mean values less than population mean. Among various genotypes CS6, CS1, CS3, CS10, CS13 and CS15 had values of regression near to unity (bi = 1) which indicated that these genotypes are stable in performance across all environments. CS7 and CS9 were responsive to favourable environments because values of regression coefficient were greater than unity (bi > 1) whereas CS2 was responsive to unfavourable environments as evident from values of regression coefficients were less than one. Results are in consonance with the inferences made by Chowdhury et al.^27^ and Senapati and Sarkar^28^.

**Table 19:** Stability parameters for days to 50 per cent flowering and days to ripe maturity.

Genotypes	Days to 50 per cent flowering			Days to ripe maturity		
	Mean	S^2^d_i_	b_i_	Mean	S^2^d_i_	b_i_
CS1	60.44	− 0.90	0.95	110.89	− 1.22	1.04
CS2	64.56	− 0.81	0.80	113.44	− 0.82	1.08
CS3	56.33	0.17	0.95	106.78	− 0.59	1.10
CS4	65.67	− 0.90	0.97	118.22	0.07	1.03
CS5	70.89	− 0.78	0.98	120.44	0.71	0.77
CS6	62.56	− 0.80	1.02	114.44	− 0.39	1.13
CS7	52.89	− 0.56	1.17	104.89	− 0.70	1.34
CS8	73.56	− 0.58	1.19	125.44	− 0.48	0.87
CS9	53.11	− 0.90	1.19	111.11	− 1.36	1.15
CS10	60.89	− 0.25	1.02	112.78	− 1.33	0.95
CS11	70.22	− 0.79	1.14	120.44	− 0.86	0.82
CS12	65.89	− 0.80	1.02	115.67	− 1.23	1.18
CS13	55.11	− 0.56	1.05	106.00	− 1.10	1.56
CS14	64.33	− 0.29	0.81	114.89	− 0.14	0.67
CS15	55.22	− 0.90	1.02	109.44	− 1.10	1.16
CS16	63.33	0.15	0.82	119.33	− 1.00	0.58
CS17	70.33	− 0.23	1.09	124.00	1.28	0.84
CS18	64.33	2.27	0.95	118.56	− 1.14	0.82
CS19	65.78	0.25	0.95	118.78	− 0.87	0.95
DKC-8	67.89	− 0.59	0.98	115.78	− 1.18	0.97
Mean	63.17			115.07		

bi, regression coefficient; S^2^d_i_ , squared deviation from linearity of regression.

##### Days to ripe maturity

Deviation from linear regression (S^2^di) was non-significant for all the genotypes indicating less contribution of linear regression toward G × E interaction (Table 20). Genotypes CS7, CS13, CS15, CS3, CS10, CS14, CS1, CS9, CS2 and CS6 were early in ripe maturity. Among various landraces CS2, CS1, CS10 and CS3 had regression coefficient near to unity which indicated that these genotypes were considered as stable across all environments. Whereas, CS6, CS7, CS9, CS13 and CS15 had regression coefficient greater than1 indicated that specifically adapted to favourable environments. CS8, and CS14 were highly responsive to unfavourable environments as evident from bi value less than one.

**Table 20:** Stability parameters for plant height (cm), Number of ripe fruits and ripe fruit yield (g).

Genotypes	Plant height (cm)			Number of fruits plant^−1^		
	Mean	Mean	S^2^d_i_	Mean	b_i_	S^2^d_i_
CS1	91.68	1.306	− 0.53	53.10	0.55	1.70
CS2	81.14	0.945	− 0.50	60.30	0.80	− 0.57
CS3	62.06	0.973	− 0.54	75.48	0.93	− 1.72
CS4	71.47	0.997	− 0.54	48.10	0.89	− 0.28
CS5	78.01	0.125	− 0.44	30.34	0.73	− 1.51
CS6	89.44	0.948	− 0.53	85.51	0.99	5.55
CS7	83.23	0.670	− 0.53	63.37	0.53	− 0.68
CS8	93.89	0.20	− 0.03	38.08	1.14	0.09
CS9	66.43	1.04	− 0.32	64.50	1.21	− 1.12
CS10	100.87	0.96	− 0.54	64.77	0.96	− 0.75
CS11	90.71	1.16	− 0.50	59.56	1.28	0.31
CS12	69.64	0.69	− 0.10	47.98	1.72	− 1.67
CS13	78.15	1.24	− 0.54	82.9	1.00	− 1.49
CS14	68.09	1.57	− 0.29	62.97	0.74	− 1.72
CS15	96.08	1.00	− 0.19	61.78	0.81	− 1.19
CS16	62.54	1.36	− 0.11	63.10	1.64	− 1.58
CS17	82.02	1.06	− 0.54	45.24	1.27	− 0.17
CS18	72.81	1.72	− 0.53	83.83	0.91	− 1.75
CS19	88.86	1.07	0.32	74.20	1.03	0.21
DKC-8	63.08	0.98	− 0.29	71.53	0.70	− 1.25
Mean	79.51			61.83		

bi, regression coefficient; S^2^d_i_ , squared deviation from linearity of regression.

##### Plant height (cm)

The data in Table 20 showed that deviation from linear regression (S^2^di) was non-significant for all the genotypes indicating less contribution of linear regression toward G × E interaction. Genotypes CS10, CS15, CS8, CS1, CS5, CS11, CS19 CS7, CS17 and CS2 had mean value greater than that of population mean. Among various Genotypes CS1, CS2, CS6, CS10, CS15, CS17 and CS19 had regression coefficient near to unity indicated that these genotypes well adapted to across all the environments for this trait. CS2, CS7 and CS8 were responsive to unfavourable environment as evident from bi value is less than 1. Whereas, CS11 exhibit regression coefficient greater than 1 which indicated that specifically adapted to favourable environments for this characteristic.

##### Number of ripe fruits plant^−1^

Deviation from linear regression (S^2^di) was non-significant for all the genotypes indicating less contribution of linear regression toward G × E interaction. Genotypes CS3, CS6, CS7, CS9, CS10, CS13, CS14, CS16, CS18, CS19 and DKC-8 had mean values greater than population mean as presented in Table 20. Genotypes CS13, CS6, CS10 CS19, CS3 and CS18 exhibited regression coefficients near to unity which indicated that these landraces can performed uniformly equal in all the environments. Genotypes CS9 and CS16 were responsive to favourable environments because bi value was greater than 1. Whereas, genotypes CS7, CS14 and DKC-8 were responsive to unfavourable environments because value of regression coefficient was less than one.

##### Average ripe fruit weight (g)

The perusal of data (Table 21) indicated that deviation from linear regression was significant for all the genotypes. Genotypes CS5, CS8, CS9, CS10, CS12, CS15, CS16 and CS18 had mean values greater than population mean and exhibited value of regression greater or less than 1 but their performance was unpredictable over environments because of deviation from linear regression was significant which indicated that average ripe fruit weight was influenced more by genetic components than environmental component.

**Table 21:** Stability parameters for average ripe fruit weight (g) and ripe fruit yield per plant (g).

Genotypes	Average ripe fruit weight (g)			Ripe fruit yield per plant (g)		
	Mean	b_i_	S^2^d_i_	Mean	b_i_	S^2^d_i_
CS1	1.99	0.43	− 0.004*	105.93	0.35	− 22.16
CS2	2.11	0.34	− 0.003*	127.12	0.49	− 11.49
CS3	2.40	1.37	− 0.004*	181.04	0.92	− 27.18
CS4	2.64	1.49	− 0.003*	126.98	0.83	− 24.23
CS5	3.57	3.36	− 0.004*	108.63	1.00	− 26.21
CS6	2.70	0.59	− 0.004*	231.12	0.64	− 3.65
CS7	1.94	0.30	− 0.004*	122.87	0.33	− 21.76
CS8	4.66	1.11	− 0.004*	177.50	1.46	13.12
CS9	5.01	− 0.04	− 0.004*	323.35	1.49	− 9.18
CS10	3.85	1.83	− 0.002*	249.24	0.88	11.85
CS11	2.90	0.05	− 0.002*	172.48	0.93	− 25.89
CS12	3.36	1.95	− 0.004*	161.61	1.74	− 25.15
CS13	3.04	2.72	− 0.004*	252.76	1.58	− 20.95
CS14	2.78	0.24	− 0.004*	175.01	0.56	− 27.90
CS15	4.72	1.42	− 0.004*	292.02	1.27	− 18.41
CS16	3.66	1.28	− 0.004*	231.60	1.77	− 25.66
CS17	2.72	0.96	− 0.004*	123.19	1.00	− 6.10
CS18	3.34	− 0.08	− 0.004*	279.99	0.73	− 27.95
CS19	2.76	0.42	− 0.004*	205.17	1.48	− 22.30
DKC-8	2.74	0.27	− 0.004*	195.87	0.54	− 21.46
Mean	3.14			192.17		

bi, regression coefficient; S^2^d_i_, squared deviation from linearity of regression.

##### Ripe fruit yield per plant (g)

Deviation from linear regression (S^2^di) was non-significant for all the genotypes indicating less contribution of linear regression toward G × E interaction. Landraces CS10, CS6 and CS19 exhibited regression coefficients near to unity which indicated that these landraces can performe uniformly equal in all the environments (Table 21). Genotypes CS9, CS13, CS15, CS16, CS18 and DKC-8 had high mean values than that of the population mean. Results showed that CS9, CS13, CS15 and CS16 exhibited regression coefficients greater than unity (bi = 1) which indicated that specifically adapted to favourable environments. Whereas, CS18 and DKC-8 were responsive to unfavorable environments because regression coefficient was less than unity (bi = 1).

### Advantages and limitations of the study

Advantages

1. Many varieties have been recommended across agro-climate zones of Himachal Pradesh, yet the information on stability is lacking in this State. Hence, the present investigation was carried out to identify high yielding stable genotypes among various pre-adapted landraces
2. In this study, The Local chilli landraces (confined to the kitchen gardens) were collected from different villages of Sirmour district of Himachal Pradesh
3. Himachal Pradesh has larger area but occupied with varieties of low yield potential. This indicates that there is need to increase average productivity of chilli in Himachal Pradesh by cultivating pre-adapted landraces because these landraces may prove are high yielding when grown *ex-situ*.
4. Chilli, being sensitive to environmental fluctuations exhibits large variations in yield. Phenotypically stable genotypes are of great importance because environmental conditions vary from season to season.
5. 1–2 genotypes produce higher yield and stable performance in both summer and rainy seasons as compared to recommended commercial variety of Himachal Pradesh i.e. DKC-8
6. In this study, Eberhart and Russell model (1940) was used which requires less area as compared to other model and provide more reliable information on stability

Limitations

1. Eberhart and Russell model^10^ does not provide independent estimation for mean performance and Environment interaction.
2. Some genotypes specifically adapted to favorable nich environments on the basis of stability parameters.
3. In this study, cultivating pre-adapted landraces were used, so recommendation may have confined to the state of Himachal Pradesh for commercial purpose.

## Conclusion

In summer seasons, genotypes CS7 (41.00) and CS9 (41.00) were earliest in flowering. Whereas in winter season, genotype CS3 (75.00) was earliest in flowering. Maximum Plant height was recorded in CS10 i.e. 102.65 cm during summer seasons and 97.20 cm during winter season. Upright fruiting habit was recorded in CS3 and DKC-8 whereas all other landraces showed dropping fruiting habit. Maximum number of ripe fruits plant^-1^ was recorded inCS6 which was statistically at par with CS18 and CS13. Average ripe fruit weight was recorded maximum in CS9 followed by CS15. Maximum ripe fruit yield plant^−1^ was recorded in CS9.

Phenotypic coefficient of variation was higher as compare d togenotypic coefficient of variation for all the parameters which showed that appearance of these traits was greatly influenced by the environment. High heritability coupled with high genetic advance was recorded for plant height followed by number of ripe fruits plant^−1^ and red ripe fruit yield plant^−1^. It is also inferred that both the seasons, summer and winter, could be utilized in evaluating landraces, which will certainly hasten the process of conventional breeding programme. But, obviously, plant height in summers and number of ripe fruits in winter will be characteristics of focus. The estimation of correlation coefficients showed that the average ripe fruit weight and number of ripe fruits plant^−1^ were positively and significantly contributing characteristics toward the ripe fruits yield plant^−1^. Although days to 50% flowering similarly contributed significantly to fruit yield, the direction of the effect was in the opposite way. Because of the earlier flowering and longer fruiting duration, it suggested that the early maturing genotypes (as observed in CS9 and CS7) would prove to be better yielders. As per path analysis, average ripe fruit weight had the highest positive direct effects on ripe fruit yield per plant followed by number of ripe fruits per plant, days to maturity (mature ripe stage). Thus, yield improvement could be achieved by direct selection for these traits. On the basis of stability analysis, Stable genotypes for different paramteres were showed in Table 22. Landraces CS2, CS1, CS10 and CS3 were stable in performance across all environments for 50 per cent flowering and ripe maturity. Genotypes CS6, CS10 and CS19 were stable in performance across all environments for plant height, number of ripe fruits and ripe fruit yield plant^-1^whereas deviation from linear regression was significant which indicated that average ripe fruit weight was influenced more by genetic components than environmental component.

**Table 22:** Stable genotypes on the basis of stability parameters.

Characters	Well adapted to all environments bi = 1	Specifically adapted favourable environments bi > 1	Specifically adapted unfavourable environments bi < 1
Days to 50% flowering	CS1, CS3, CS6, CS10, CS13, CS15	CS7 and CS9	CS2
Days to ripe maturity	CS1, CS2 and CS3	CS6, CS7, CS9, CS13 and CS15	CS8, CS10 and CS14
Plant height (cm)	CS1, CS2, CS6, CS10, CS15, CS17 and CS19	CS11	CS2, CS7 and CS8
Number of ripe fruits plant^−1^	CS13, CS3, CS6, CS10,CS19 and CS18	CS9 and CS16	, CS7, CS14 and DKC-8
Ripe fruit yield plant^−1^ (g)	CS6, CS19 and CS10	CS9, CS13, CS15 and CS16	CS18 and DKC-8

### Supplementary Information

Supplementary Information 1.

Supplementary Information 2.

## Data Availability

The datasets generated and analyzed during the current study are available in the supplementary files.
