# Supplementary material for: Mean performances, character associations and multi-environmental evaluation of chilli landraces in north western Himalayas
Source: Sci Rep. 2024 Jan 8;14:769. doi: 10.1038/s41598-024-51348-5 (PMC10774388; doi:10.1038/s41598-024-51348-5)
Supplement: Supplementary file 1 — Supplementary Information 1. [file 41598_2024_51348_MOESM1_ESM.docx]

**Raw Data**

Days to 50% flowering

|  | | | | **SUMMER SEASON 2020** | | | | | | |  | | |
| --- | --- | --- | --- | --- | --- | --- | --- | --- | --- | --- | --- | --- | --- |
|  | | | | **R1** | | | **R2** | **R3** | | | **AVERAGE** | | |
| **CS1** | | | | 50 | | | 52 | 52 | | | **51.33** | | |
| **CS2** | | | | 57 | | | 56 | 56 | | | **56.33** | | |
| **CS3** | | | | 46 | | | 46 | 47 | | | **46.33** | | |
| **CS4** | | | | 54 | | | 56 | 58 | | | **56.00** | | |
| **CS5** | | | | 61 | | | 60 | 63 | | | **61.33** | | |
| **CS6** | | | | 51 | | | 53 | 52 | | | **52.00** | | |
| **CS7** | | | | 40 | | | 42 | 40 | | | **40.67** | | |
| **CS8** | | | | 62 | | | 63 | 61 | | | **62.00** | | |
| **CS9** | | | | 41 | | | 40 | 42 | | | **41.00** | | |
| **CS10** | | | | 49 | | | 50 | 51 | | | **50.00** | | |
| **CS11** | | | | 57 | | | 58 | 62 | | | **59.00** | | |
| **CS12** | | | | 55 | | | 58 | 53 | | | **55.33** | | |
| **CS13** | | | | 48 | | | 46 | 41 | | | **45.00** | | |
| **CS14** | | | | 58 | | | 57 | 52 | | | **55.67** | | |
| **CS15** | | | | 48 | | | 45 | 42 | | | **45.00** | | |
| **CS16** | | | | 56 | | | 53 | 54 | | | **54.33** | | |
| **CS17** | | | | 59 | | | 59 | 62 | | | **60.00** | | |
| **CS18** | | | | 55 | | | 59 | 54 | | | **56.00** | | |
| **CS19** | | | | 55 | | | 59 | 57 | | | **57.00** | | |
| **DKC8** | | | | 56 | | | 58 | 59 | | | **57.67** | | |
|  | | | |  | | |  | **MEAN** | | | **53.10** | | |
| **WINTER SEASON 2020** | | | | | | | |  | | |  |  |  |
| **R1** | **R2** | | | **R3** | | | | **AVERAGE** | | |  |  |  |
| 79 | 78 | | | 80 | | | | **79.00** | | |  |  |  |
| 81 | 80 | | | 81 | | | | **80.67** | | |  |  |  |
| 76 | 75 | | | 74 | | | | **75.00** | | |  |  |  |
| 86 | 86 | | | 84 | | | | **85.33** | | |  |  |  |
| 90 | 91 | | | 91 | | | | **90.67** | | |  |  |  |
| 83 | 85 | | | 82 | | | | **83.33** | | |  |  |  |
| 76 | 77 | | | 77 | | | | **76.67** | | |  |  |  |
| 98 | 97 | | | 98 | | | | **97.67** | | |  |  |  |
| 78 | 79 | | | 75 | | | | **77.33** | | |  |  |  |
| 81 | 82 | | | 82 | | | | **81.67** | | |  |  |  |
| 93 | 94 | | | 93 | | | | **93.33** | | |  |  |  |
| 87 | 86 | | | 87 | | | | **86.67** | | |  |  |  |
| 78 | 75 | | | 76 | | | | **76.33** | | |  |  |  |
| 79 | 81 | | | 82 | | | | **80.67** | | |  |  |  |
| 77 | 76 | | | 75 | | | | **76.00** | | |  |  |  |
| 81 | 79 | | | 80 | | | | **80.00** | | |  |  |  |
| 95 | 90 | | | 92 | | | | **92.33** | | |  |  |  |
| 86 | 83 | | | 82 | | | | **83.67** | | |  |  |  |
| 84 | 85 | | | 86 | | | | **85.00** | | |  |  |  |
| 87 | 90 | | | 86 | | | | **87.67** | | |  |  |  |
|  |  | | | **MEAN** | | | | **83.45** | | |  |  |  |
| **SUMMER SEASON 2021** | | | | | | | | |  | | |  |  |
| **R1** | | **R2** | | | **R3** | | | | **AVERAGE** | | |  |  |
| 51 | | 50 | | | 52 | | | | **51.00** | | |  |  |
| 59 | | 56 | | | 55 | | | | **56.67** | | |  |  |
| 49 | | 46 | | | 48 | | | | **47.67** | | |  |  |
| 55 | | 55 | | | 57 | | | | **55.67** | | |  |  |
| 62 | | 59 | | | 61 | | | | **60.67** | | |  |  |
| 51 | | 53 | | | 53 | | | | **52.33** | | |  |  |
| 43 | | 43 | | | 38 | | | | **41.33** | | |  |  |
| 62 | | 60 | | | 61 | | | | **61.00** | | |  |  |
| 43 | | 40 | | | 40 | | | | **41.00** | | |  |  |
| 51 | | 52 | | | 50 | | | | **51.00** | | |  |  |
| 57 | | 60 | | | 58 | | | | **58.33** | | |  |  |
| 53 | | 59 | | | 55 | | | | **55.67** | | |  |  |
| 45 | | 43 | | | 44 | | | | **44.00** | | |  |  |
| 59 | | 56 | | | 55 | | | | **56.67** | | |  |  |
| 46 | | 43 | | | 45 | | | | **44.67** | | |  |  |
| 56 | | 55 | | | 56 | | | | **55.67** | | |  |  |
| 58 | | 60 | | | 58 | | | | **58.67** | | |  |  |
| 53 | | 55 | | | 52 | | | | **53.33** | | |  |  |
| 56 | | 56 | | | 54 | | | | **55.33** | | |  |  |
| 58 | | 58 | | | 59 | | | | **58.33** | | |  |  |
|  | |  | | | **MEAN** | | | | **52.95** | | |  |  |

**2. Days to Ripe Maturity**

|  | | **SUMMER SEASON 2020** | | | | | | | | |  | |
| --- | --- | --- | --- | --- | --- | --- | --- | --- | --- | --- | --- | --- |
|  | | **R1** | | | | **R2** | **R3** | | | | **AVERAGE** | |
| **CS1** | | 100 | | | | 98 | 101 | | | | **99.67** | |
| **CS2** | | 106 | | | | 100 | 100 | | | | **102.00** | |
| **CS3** | | 94 | | | | 99 | 96 | | | | **96.33** | |
| **CS4** | | 104 | | | | 108 | 109 | | | | **107.00** | |
| **CS5** | | 114 | | | | 115 | 112 | | | | **113.67** | |
| **CS6** | | 106 | | | | 102 | 99 | | | | **102.33** | |
| **CS7** | | 87 | | | | 92 | 97 | | | | **92.00** | |
| **CS8** | | 118 | | | | 117 | 117 | | | | **117.33** | |
| **CS9** | | 100 | | | | 96 | 103 | | | | **99.67** | |
| **CS10** | | 108 | | | | 104 | 101 | | | | **104.33** | |
| **CS11** | | 107 | | | | 116 | 115 | | | | **112.67** | |
| **CS12** | | 108 | | | | 102 | 102 | | | | **104.00** | |
| **CS13** | | 91 | | | | 90 | 91 | | | | **90.67** | |
| **CS14** | | 105 | | | | 109 | 108 | | | | **107.33** | |
| **CS15** | | 93 | | | | 101 | 98 | | | | **97.33** | |
| **CS16** | | 110 | | | | 114 | 115 | | | | **113.00** | |
| **CS17** | | 115 | | | | 116 | 112 | | | | **114.33** | |
| **CS18** | | 110 | | | | 113 | 109 | | | | **110.67** | |
| **CS19** | | 107 | | | | 110 | 109 | | | | **108.67** | |
| **DKC8** | | 106 | | | | 105 | 108 | | | | **106.33** | |
|  | |  | | | |  | **MEAN** | | | | **104.97** | |
| **WINTER SEASON 2020** | | | | | | | | |  | | |  |
| **R1** | **R2** | | | **R3** | | | | | **AVERAGE** | | |  |
| 132 | 135 | | | 136 | | | | | **134.33** | | |  |
| 137 | 134 | | | 136 | | | | | **135.67** | | |  |
| 130 | 129 | | | 129 | | | | | **129.33** | | |  |
| 139 | 140 | | | 139 | | | | | **139.33** | | |  |
| 135 | 137 | | | 137 | | | | | **136.33** | | |  |
| 137 | 138 | | | 138 | | | | | **137.67** | | |  |
| 132 | 133 | | | 132 | | | | | **132.33** | | |  |
| 143 | 144 | | | 143 | | | | | **143.33** | | |  |
| 134 | 134 | | | 136 | | | | | **134.67** | | |  |
| 129 | 131 | | | 131 | | | | | **130.33** | | |  |
| 136 | 138 | | | 138 | | | | | **137.33** | | |  |
| 139 | 142 | | | 139 | | | | | **140.00** | | |  |
| 137 | 138 | | | 139 | | | | | **138.00** | | |  |
| 128 | 128 | | | 130 | | | | | **128.67** | | |  |
| 133 | 134 | | | 133 | | | | | **133.33** | | |  |
| 132 | 131 | | | 131 | | | | | **131.33** | | |  |
| 141 | 142 | | | 141 | | | | | **141.33** | | |  |
| 135 | 134 | | | 137 | | | | | **135.33** | | |  |
| 138 | 139 | | | 138 | | | | | **138.33** | | |  |
| 137 | 136 | | | 134 | | | | | **135.67** | | |  |
|  |  | | | **MEAN** | | | | | **135.63** | | |  |
|  | | | **SUMMER SEASON 2021** | | | | | | |  |  |  |
| **R1** | | | **R2** | | **R3** | | | **AVERAGE** | |  |  |  |
| 98 | | | 98 | | 100 | | | **98.67** | |  |  |  |
| 105 | | | 101 | | 102 | | | **102.67** | |  |  |  |
| 93 | | | 97 | | 94 | | | **94.67** | |  |  |  |
| 106 | | | 110 | | 109 | | | **108.33** | |  |  |  |
| 112 | | | 111 | | 111 | | | **111.33** | |  |  |  |
| 105 | | | 103 | | 102 | | | **103.33** | |  |  |  |
| 89 | | | 90 | | 92 | | | **90.33** | |  |  |  |
| 115 | | | 116 | | 116 | | | **115.67** | |  |  |  |
| 98 | | | 99 | | 100 | | | **99.00** | |  |  |  |
| 106 | | | 102 | | 103 | | | **103.67** | |  |  |  |
| 110 | | | 113 | | 111 | | | **111.33** | |  |  |  |
| 105 | | | 101 | | 103 | | | **103.00** | |  |  |  |
| 89 | | | 90 | | 89 | | | **89.33** | |  |  |  |
| 110 | | | 108 | | 108 | | | **108.67** | |  |  |  |
| 96 | | | 98 | | 99 | | | **97.67** | |  |  |  |
| 115 | | | 114 | | 112 | | | **113.67** | |  |  |  |
| 116 | | | 118 | | 115 | | | **116.33** | |  |  |  |
| 109 | | | 112 | | 108 | | | **109.67** | |  |  |  |
| 107 | | | 110 | | 111 | | | **109.33** | |  |  |  |
| 105 | | | 107 | | 104 | | | **105.33** | |  |  |  |
|  | | |  | | **MEAN** | | | **104.60** | |  |  |  |

**Plant Height**

|  | **SUMMER SEASON 2020** | | |  |
| --- | --- | --- | --- | --- |
|  | **R1** | **R2** | **R3** | **AVERAGE** |
| **CS1** | 95.42 | 94.31 | 93.55 | **94.43** |
| **CS2** | 82.13 | 83.22 | 84.32 | **83.22** |
| **CS3** | 64.64 | 63.81 | 63.74 | **64.06** |
| **CS4** | 73.43 | 72.54 | 74.62 | **73.53** |
| **CS5** | 79.94 | 78.57 | 76.95 | **78.49** |
| **CS6** | 91.12 | 90.49 | 92.19 | **91.27** |
| **CS7** | 83.95 | 84.69 | 84.93 | **84.52** |
| **CS8** | 94.76 | 93.36 | 93.25 | **93.79** |
| **CS9** | 69.87 | 67.89 | 68.9 | **68.89** |
| **CS10** | 103.52 | 102.92 | 101.94 | **102.79** |
| **CS11** | 92.98 | 93.24 | 93.44 | **93.22** |
| **CS12** | 71.64 | 70.9 | 71.98 | **71.51** |
| **CS13** | 80.96 | 81.24 | 79.96 | **80.72** |
| **CS14** | 71.97 | 70.98 | 69.87 | **70.94** |
| **CS15** | 98.94 | 98.76 | 97.92 | **98.54** |
| **CS16** | 65.9 | 64.74 | 63.98 | **64.87** |
| **CS17** | 84.31 | 83.88 | 84.32 | **84.17** |
| **CS18** | 75.76 | 76.55 | 76.42 | **76.24** |
| **CS19** | 90.39 | 91.25 | 89.58 | **90.41** |
| **DKC8** | 65.49 | 64.97 | 65.82 | **65.43** |
|  |  |  | **MEAN** | **81.55** |

|  | **WINTER SEASON 2020** | | |  |
| --- | --- | --- | --- | --- |
|  | **R1** | **R2** | **R3** | **AVERAGE** |
|  | 87.15 | 86.14 | 86.78 | **86.69** |
|  | 78.25 | 76 | 78.34 | **77.53** |
|  | 58.12 | 57.32 | 59.56 | **58.33** |
|  | 68.45 | 67.64 | 66.87 | **67.65** |
|  | 78.32 | 78.47 | 75.83 | **77.54** |
|  | 87.23 | 85.87 | 84.45 | **85.85** |
|  | 81.23 | 81.45 | 79.32 | **80.67** |
|  | 94.47 | 93.21 | 91.65 | **93.11** |
|  | 62.56 | 61.89 | 62.93 | **62.46** |
|  | 97.34 | 96.76 | 97.49 | **97.20** |
|  | 83.24 | 82.36 | 93.25 | **86.28** |
|  | 68.34 | 65.78 | 66.96 | **67.03** |
|  | 72.05 | 74.23 | 73.9 | **73.39** |
|  | 61.23 | 62.56 | 62.45 | **62.08** |
|  | 92.68 | 91.35 | 92.76 | **92.26** |
|  | 58.54 | 57.26 | 56.12 | **57.31** |
|  | 79.11 | 76.68 | 78.12 | **77.97** |
|  | 67.36 | 66.2 | 65.12 | **66.23** |
|  | 85.09 | 82.05 | 87.03 | **84.72** |
|  | 59.19 | 60.37 | 58.49 | **59.35** |
|  |  |  | **MEAN** | **75.68** |

| **SUMMER SEASON 2021** | | |  |
| --- | --- | --- | --- |
| **R1** | **R2** | **R3** | **AVERAGE** |
| 94.98 | 94.21 | 92.62 | **93.94** |
| 81.92 | 83.52 | 82.56 | **82.67** |
| 63.38 | 64.69 | 63.24 | **63.77** |
| 72.83 | 72.94 | 73.87 | **73.21** |
| 79.54 | 77.53 | 76.92 | **78.00** |
| 90.12 | 90.49 | 93.02 | **91.21** |
| 84.91 | 83.76 | 84.86 | **84.51** |
| 93.66 | 95.86 | 94.75 | **94.76** |
| 67.49 | 67.79 | 68.55 | **67.94** |
| 101.98 | 103.12 | 102.76 | **102.62** |
| 91.92 | 93.59 | 92.33 | **92.61** |
| 69.64 | 70.65 | 70.88 | **70.39** |
| 81.96 | 79.64 | 79.41 | **80.34** |
| 72.23 | 70.48 | 71.05 | **71.25** |
| 97.18 | 99.05 | 96.08 | **97.44** |
| 66.59 | 64.14 | 65.63 | **65.45** |
| 84.46 | 82.36 | 84.92 | **83.91** |
| 76.13 | 75.35 | 76.38 | **75.95** |
| 90.83 | 92.13 | 91.38 | **91.45** |
| 64.19 | 63.67 | 65.52 | **64.46** |
|  |  | **MEAN** | **81.29** |

**Mean performance of chilli genotypes for fruiting habit and fruit blossom end shape:**

| **Genotypes** | **Fruiting habit** | **Fruit blossom end shape** |
| --- | --- | --- |
| **CS1** | Drooping | Pointed |
| **CS2** | Drooping | Pointed |
| **CS3** | Upright | Pointed |
| **CS4** | Drooping | Pointed |
| **CS5** | Drooping | Pointed |
| **CS6** | Drooping | Pointed |
| **CS7** | Drooping | Pointed |
| **CS8** | Drooping | Pointed |
| **CS9** | Drooping | Pointed |
| **CS10** | Drooping | Pointed |
| **CS11** | Drooping | Pointed |
| **CS12** | Drooping | Pointed |
| **CS13** | Drooping | Pointed |
| **CS14** | Drooping | Pointed |
| **CS15** | Drooping | Pointed |
| **CS16** | Drooping | Pointed |
| **CS17** | Drooping | Pointed |
| **CS18** | Drooping | Pointed |
| **CS19** | Drooping | Pointed |
| **DKC-8** | Upright | Pointed |

**Number of Ripe Fruits Per Plant**

|  | **SUMMER SEASON 2020** | | |  |
| --- | --- | --- | --- | --- |
|  | **R1** | **R2** | **R3** | **AVERAGE** |
| **CS1** | 56.23 | 54.15 | 56.43 | **55.60** |
| **CS2** | 61.50 | 61.56 | 60.50 | **61.19** |
| **CS3** | 79.18 | 78.63 | 74.03 | **77.28** |
| **CS4** | 51.30 | 45.47 | 50.45 | **49.07** |
| **CS5** | 34.50 | 31.77 | 30.42 | **32.23** |
| **CS6** | 89.78 | 90.24 | 86.74 | **88.92** |
| **CS7** | 64.50 | 66.48 | 64.69 | **65.22** |
| **CS8** | 42.40 | 42.44 | 39.52 | **41.45** |
| **CS9** | 65.50 | 66.48 | 67.34 | **66.44** |
| **CS10** | 65.60 | 68.21 | 65.54 | **66.45** |
| **CS11** | 58.34 | 61.23 | 64.04 | **61.20** |
| **CS12** | 50.12 | 51.89 | 52.05 | **51.35** |
| **CS13** | 86.60 | 82.78 | 84.56 | **84.65** |
| **CS14** | 65.40 | 66.20 | 62.40 | **64.67** |
| **CS15** | 58.34 | 66.00 | 67.73 | **64.02** |
| **CS16** | 64.70 | 65.54 | 68.46 | **66.23** |
| **CS17** | 46.40 | 47.76 | 46.79 | **46.98** |
| **CS18** | 86.20 | 86.31 | 84.54 | **85.68** |
| **CS19** | 78.48 | 75.87 | 77.67 | **77.34** |
| **DKC8** | 74.34 | 72.56 | 70.53 | **72.48** |
|  |  |  | **MEAN** | **63.92** |

| **WINTER SEASON 2020** | | | | | |  | |
| --- | --- | --- | --- | --- | --- | --- | --- |
| **R1** | | **R2** | | **R3** | | **AVERAGE** | |
| 50.20 | | 51.00 | | 50.40 | | **50.53** | |
| 56.20 | | 57.00 | | 56.90 | | **56.70** | |
| 71.20 | | 72.00 | | 70.60 | | **71.27** | |
| 44.30 | | 44.90 | | 43.20 | | **44.13** | |
| 26.00 | | 27.80 | | 27.20 | | **27.00** | |
| 82.30 | | 81.50 | | 83.00 | | **82.27** | |
| 60.20 | | 61.00 | | 61.60 | | **60.93** | |
| 32.00 | | 33.00 | | 33.60 | | **32.87** | |
| 59.00 | | 57.00 | | 61.20 | | **59.07** | |
| 62.40 | | 63.38 | | 62.20 | | **62.66** | |
| 53.36 | | 54.33 | | 53.70 | | **53.80** | |
| 40.20 | | 41.00 | | 39.46 | | **40.22** | |
| 79.00 | | 78.00 | | 78.20 | | **78.40** | |
| 60.20 | | 59.00 | | 59.60 | | **59.60** | |
| 60.20 | | 56.00 | | 58.00 | | **58.07** | |
| 54.00 | | 56.00 | | 57.00 | | **55.67** | |
| 39.20 | | 39.40 | | 40.00 | | **39.53** | |
| 80.20 | | 79.00 | | 79.90 | | **79.70** | |
| 65.20 | | 66.30 | | 64.20 | | **65.23** | |
| 69.46 | | 68.40 | | 67.30 | | **68.39** | |
|  | |  | | **MEAN** | | **57.30** | |
| **SUMMER SEASON 2021** | | | | |  | |  |
| **R1** | **R2** | | **R3** | | **AVERAGE** | |  |
| 54.18 | 53.12 | | 52.21 | | **53.17** | |  |
| 62.36 | 64.20 | | 62.46 | | **63.01** | |  |
| 83.28 | 77.24 | | 73.13 | | **77.88** | |  |
| 54.23 | 46.68 | | 52.39 | | **51.10** | |  |
| 33.44 | 31.65 | | 30.24 | | **31.78** | |  |
| 86.40 | 87.45 | | 82.18 | | **85.34** | |  |
| 60.25 | 63.14 | | 68.44 | | **63.94** | |  |
| 45.33 | 38.43 | | 36.03 | | **39.93** | |  |
| 68.68 | 68.42 | | 66.87 | | **67.99** | |  |
| 62.42 | 64.37 | | 68.78 | | **65.19** | |  |
| 59.34 | 62.98 | | 68.72 | | **63.68** | |  |
| 48.48 | 56.41 | | 52.22 | | **52.37** | |  |
| 88.21 | 83.54 | | 85.43 | | **85.73** | |  |
| 64.00 | 63.00 | | 66.90 | | **64.63** | |  |
| 61.92 | 62.76 | | 65.04 | | **63.24** | |  |
| 68.46 | 69.42 | | 64.32 | | **67.40** | |  |
| 45.40 | 51.24 | | 50.98 | | **49.21** | |  |
| 85.41 | 89.68 | | 83.21 | | **86.10** | |  |
| 79.56 | 78.91 | | 81.58 | | **80.02** | |  |
| 70.72 | 75.18 | | 75.28 | | **73.73** | |  |
|  |  | | **MEAN** | | **64.27** | |  |

**Average ripe fruit weight**

|  | | **SUMMER SEASON 2020** | | | | |  | | |
| --- | --- | --- | --- | --- | --- | --- | --- | --- | --- |
|  | | **R1** | | **R2** | **R3** | | **AVERAGE** | | |
| **CS1** | | 1.98 | | 2.02 | 1.97 | | **1.99** | | |
| **CS2** | | 2.13 | | 2.01 | 2.15 | | **2.10** | | |
| **CS3** | | 2.34 | | 2.46 | 2.56 | | **2.45** | | |
| **CS4** | | 2.65 | | 2.78 | 2.68 | | **2.70** | | |
| **CS5** | | 3.46 | | 3.89 | 3.65 | | **3.67** | | |
| **CS6** | | 2.76 | | 2.65 | 2.69 | | **2.70** | | |
| **CS7** | | 1.92 | | 1.98 | 1.96 | | **1.95** | | |
| **CS8** | | 4.66 | | 4.65 | 4.76 | | **4.69** | | |
| **CS9** | | 4.98 | | 5.05 | 4.99 | | **5.01** | | |
| **CS10** | | 3.86 | | 3.98 | 3.95 | | **3.93** | | |
| **CS11** | | 2.98 | | 2.94 | 2.87 | | **2.93** | | |
| **CS12** | | 3.40 | | 3.11 | 3.71 | | **3.41** | | |
| **CS13** | | 3.11 | | 3.20 | 3.05 | | **3.12** | | |
| **CS14** | | 2.76 | | 2.89 | 2.67 | | **2.77** | | |
| **CS15** | | 4.79 | | 4.68 | 4.82 | | **4.76** | | |
| **CS16** | | 3.45 | | 3.78 | 3.87 | | **3.70** | | |
| **CS17** | | 2.87 | | 2.45 | 2.87 | | **2.73** | | |
| **CS18** | | 3.40 | | 3.21 | 3.40 | | **3.34** | | |
| **CS19** | | 2.68 | | 2.73 | 2.96 | | **2.79** | | |
| **DKC8** | | 2.72 | | 2.74 | 2.76 | | **2.74** | | |
|  | |  | |  | **MEAN** | | **3.17** | | |
| **WINTER SEASON 2020** | | | | | |  | | |  |
| **R1** | **R2** | | **R3** | | | **AVERAGE** | | |  |
| 1.94 | 1.97 | | 1.99 | | | **1.97** | | |  |
| 2.10 | 2.05 | | 2.11 | | | **2.09** | | |  |
| 2.38 | 2.28 | | 2.25 | | | **2.30** | | |  |
| 2.55 | 2.58 | | 2.47 | | | **2.53** | | |  |
| 3.24 | 3.38 | | 3.40 | | | **3.34** | | |  |
| 2.68 | 2.59 | | 2.72 | | | **2.66** | | |  |
| 1.89 | 1.94 | | 1.92 | | | **1.92** | | |  |
| 4.47 | 4.59 | | 4.68 | | | **4.58** | | |  |
| 4.98 | 5.05 | | 5.02 | | | **5.02** | | |  |
| 3.72 | 3.68 | | 3.75 | | | **3.72** | | |  |
| 2.86 | 2.92 | | 2.89 | | | **2.89** | | |  |
| 3.19 | 3.23 | | 3.26 | | | **3.23** | | |  |
| 2.84 | 2.87 | | 2.86 | | | **2.86** | | |  |
| 2.79 | 2.73 | | 2.77 | | | **2.76** | | |  |
| 4.57 | 4.68 | | 4.63 | | | **4.63** | | |  |
| 3.39 | 3.68 | | 3.66 | | | **3.58** | | |  |
| 2.50 | 2.65 | | 2.82 | | | **2.66** | | |  |
| 3.38 | 3.34 | | 3.32 | | | **3.35** | | |  |
| 2.76 | 2.62 | | 2.82 | | | **2.73** | | |  |
| 2.74 | 2.70 | | 2.72 | | | **2.72** | | |  |
|  |  | | **MEAN** | | | **3.08** | | |  |
| **SUMMER SEASON 2021** | | | | | |  | |  |  |
| **R1** | **R2** | | **R3** | | | **AVERAGE** | |  |  |
| 2.02 | 2.06 | | 2.00 | | | **2.03** | |  |  |
| 2.17 | 2.06 | | 2.19 | | | **2.14** | |  |  |
| 2.31 | 2.41 | | 2.60 | | | **2.44** | |  |  |
| 2.61 | 2.76 | | 2.66 | | | **2.68** | |  |  |
| 3.50 | 3.93 | | 3.69 | | | **3.71** | |  |  |
| 2.81 | 2.69 | | 2.73 | | | **2.74** | |  |  |
| 1.89 | 1.95 | | 1.99 | | | **1.94** | |  |  |
| 4.69 | 4.62 | | 4.79 | | | **4.70** | |  |  |
| 5.01 | 5.08 | | 4.96 | | | **5.02** | |  |  |
| 3.83 | 3.93 | | 3.91 | | | **3.89** | |  |  |
| 2.93 | 2.78 | | 2.90 | | | **2.87** | |  |  |
| 3.44 | 3.15 | | 3.75 | | | **3.45** | |  |  |
| 3.15 | 3.21 | | 3.10 | | | **3.15** | |  |  |
| 2.78 | 2.91 | | 2.71 | | | **2.80** | |  |  |
| 4.78 | 4.72 | | 4.85 | | | **4.78** | |  |  |
| 3.48 | 3.76 | | 3.91 | | | **3.72** | |  |  |
| 2.92 | 2.49 | | 2.92 | | | **2.78** | |  |  |
| 3.42 | 3.22 | | 3.38 | | | **3.34** | |  |  |
| 2.63 | 2.75 | | 2.92 | | | **2.77** | |  |  |
| 2.73 | 2.79 | | 2.74 | | | **2.75** | |  |  |
|  |  | | **MEAN** | | | **3.18** | |  |  |

**Ripe fruit yield per plant**

|  | **SUMMER SEASON 2020** | | | | | | | | |
| --- | --- | --- | --- | --- | --- | --- | --- | --- | --- |
| **R1** | **R2** | | | | **R3** | | **AVERAGE** | | |
| 111.34 | 109.38 | | | | 111.17 | | **110.63** | | |
| 131.00 | 123.74 | | | | 130.08 | | **128.27** | | |
| 185.28 | 193.43 | | | | 189.52 | | **189.41** | | |
| 135.95 | 126.41 | | | | 135.21 | | **132.52** | | |
| 119.37 | 123.59 | | | | 111.03 | | **118.00** | | |
| 247.79 | 239.14 | | | | 233.33 | | **240.09** | | |
| 123.84 | 131.63 | | | | 126.79 | | **127.42** | | |
| 197.58 | 197.35 | | | | 188.12 | | **194.35** | | |
| 326.19 | 335.72 | | | | 336.03 | | **332.65** | | |
| 253.22 | 271.48 | | | | 258.88 | | **261.19** | | |
| 173.85 | 180.02 | | | | 183.79 | | **179.22** | | |
| 170.41 | 161.38 | | | | 193.11 | | **174.96** | | |
| 269.33 | 264.90 | | | | 257.91 | | **264.04** | | |
| 180.50 | 191.32 | | | | 166.61 | | **179.48** | | |
| 279.45 | 308.88 | | | | 326.46 | | **304.93** | | |
| 223.22 | 247.74 | | | | 264.94 | | **245.30** | | |
| 133.17 | 117.01 | | | | 134.29 | | **128.16** | | |
| 293.08 | 277.06 | | | | 287.44 | | **285.86** | | |
| 210.33 | 207.13 | | | | 229.90 | | **215.78** | | |
| 202.20 | 198.81 | | | | 194.66 | | **198.56** | | |
|  |  | | | | **MEAN** | | **200.54** | | |
|  | **WINTER SEASON 2020** | | | | | | |  |  |
| **R1** | **R2** | | **R3** | | | **AVERAGE** | |  |  |
| 97.39 | 100.47 | | 100.30 | | | **99.38** | |  |  |
| 118.02 | 116.85 | | 120.06 | | | **118.31** | |  |  |
| 169.46 | 164.16 | | 158.85 | | | **164.16** | |  |  |
| 112.97 | 115.84 | | 106.70 | | | **111.84** | |  |  |
| 84.24 | 93.96 | | 92.48 | | | **90.23** | |  |  |
| 220.56 | 211.09 | | 225.76 | | | **219.14** | |  |  |
| 113.78 | 118.34 | | 118.27 | | | **116.80** | |  |  |
| 143.04 | 151.47 | | 157.25 | | | **150.59** | |  |  |
| 293.82 | 287.85 | | 307.22 | | | **296.30** | |  |  |
| 232.13 | 233.24 | | 233.25 | | | **232.87** | |  |  |
| 152.61 | 158.64 | | 155.19 | | | **155.48** | |  |  |
| 128.24 | 132.43 | | 128.64 | | | **129.77** | |  |  |
| 224.36 | 223.86 | | 223.65 | | | **223.96** | |  |  |
| 167.96 | 161.07 | | 165.09 | | | **164.71** | |  |  |
| 275.11 | 262.08 | | 268.54 | | | **268.58** | |  |  |
| 183.06 | 206.08 | | 208.62 | | | **199.25** | |  |  |
| 98.00 | 104.41 | | 112.80 | | | **105.07** | |  |  |
| 271.08 | 263.86 | | 265.27 | | | **266.73** | |  |  |
| 179.95 | 173.71 | | 181.04 | | | **178.23** | |  |  |
| 190.32 | 184.68 | | 183.06 | | | **186.02** | |  |  |
|  |  | | **MEAN** | | | **173.87** | |  |  |
|  | | **SUMMER SEASON 2021** | | | | | | |  |
| **R1** | | **R2** | | **R3** | | **AVERAGE** | | |  |
| 109.44 | | 109.43 | | 104.42 | | **107.76** | | |  |
| 135.32 | | 132.25 | | 136.79 | | **134.79** | | |  |
| 192.38 | | 186.15 | | 190.14 | | **189.55** | | |  |
| 141.54 | | 128.84 | | 139.36 | | **136.58** | | |  |
| 117.04 | | 124.38 | | 111.59 | | **117.67** | | |  |
| 242.78 | | 235.24 | | 224.35 | | **234.13** | | |  |
| 113.87 | | 123.12 | | 136.20 | | **124.40** | | |  |
| 212.60 | | 177.55 | | 172.58 | | **187.58** | | |  |
| 344.09 | | 347.57 | | 331.68 | | **341.11** | | |  |
| 239.07 | | 252.97 | | 268.93 | | **253.66** | | |  |
| 173.87 | | 175.08 | | 199.29 | | **182.75** | | |  |
| 166.77 | | 177.69 | | 195.83 | | **180.10** | | |  |
| 277.86 | | 268.16 | | 264.83 | | **270.29** | | |  |
| 177.92 | | 183.33 | | 181.30 | | **180.85** | | |  |
| 295.98 | | 296.23 | | 315.44 | | **302.55** | | |  |
| 238.24 | | 261.02 | | 251.49 | | **250.25** | | |  |
| 132.57 | | 127.59 | | 148.86 | | **136.34** | | |  |
| 292.10 | | 288.77 | | 281.25 | | **287.37** | | |  |
| 209.24 | | 217.00 | | 238.21 | | **221.49** | | |  |
| 193.07 | | 209.75 | | 206.27 | | **203.03** | | |  |
|  | |  | | **MEAN** | | **202.11** | | |  |
