# Supplementary material for: Mean performances, character associations and multi-environmental evaluation of chilli landraces in north western Himalayas
Source: Sci Rep. 2024 Jan 8;14:769. doi: 10.1038/s41598-024-51348-5 (PMC10774388; doi:10.1038/s41598-024-51348-5)
Supplement: Supplementary file 2 — Supplementary Information 2. [file 41598_2024_51348_MOESM2_ESM.docx]

**APPENDIX - I**

**Mean meteorological data during the research period (January 2020 to July 2020)**

| **Month, Year** | **Temperature**  **(°C)** | | | **Relative Humidity**  **(%)** | **Total**  **Precipitation (mm)** |
| --- | --- | --- | --- | --- | --- |
|  | **Maximum** | **Minimum** | **Mean** |  |  |
| **January, 2020** | 20.23 | 9.87 | 15.60 | 86.90 | 11.30 |
| **February, 2020** | 21.29 | 11.30 | 16.75 | 85.65 | 4.08 |
| **March, 2020** | 28.65 | 15.45 | 21.40 | 67.00 | 13.56 |
| **May, 2020** | 35.45 | 21.13 | 27.70 | 64.00 | 12.30 |
| **June, 2020** | 36.54 | 23.30 | 26.89 | 74.00 | 14.69 |
| **July, 2020** | 37.50 | 23.69 | 24.40 | 89.54 | 412.67 |

**Mean meteorological data during the research period (September 2020 to April 2021)**

| **Month, Year** | **Temperature**  **(°C)** | | | **Relative Humidity**  **(%)** | **Total**  **Precipitation (mm)** |
| --- | --- | --- | --- | --- | --- |
|  | **Maximum** | **Minimum** | **Mean** |  |  |
| **September, 2020** | 32.68 | 20.32 | 23.75 | 92.00 | 15.20 |
| **October, 2020** | 31.43 | 18.39 | 22.65 | 89.60 | - |
| **November, 2020** | 27.68 | 12.47 | 18.54 | 54.38 | - |
| **December, 2020** | 22.04 | 10.54 | 16.27 | 47.56 | 4.29 |
| **January, 2021** | 20.43 | 10.70 | 15.60 | 82.90 | 11.34 |
| **February, 2021** | 21.26 | 12.40 | 16.75 | 80.65 | 4.03 |
| **March, 2021** | 28.97 | 17.85 | 22.47 | 63.00 | 14.56 |
| **April, 2021** | 36.45 | 20.43 | 27.79 | 65.00 | 12.38 |

**Mean meteorological data during the research period (January 2021 to July 2021)**

| **Month, Year** | **Temperature**  **(°C)** | | | **Relative Humidity**  **(%)** | **Total**  **Precipitation (mm)** |
| --- | --- | --- | --- | --- | --- |
|  | **Maximum** | **Minimum** | **Mean** |  |  |
| **January, 2021** | 20.43 | 10.70 | 15.60 | 82.90 | 11.34 |
| **February, 2021** | 21.26 | 12.40 | 16.75 | 80.65 | 4.03 |
| **March, 2021** | 28.97 | 17.85 | 22.47 | 63.00 | 14.56 |
| **May, 2021** | 36.45 | 20.43 | 27.79 | 65.00 | 12.38 |
| **June, 2021** | 34.54 | 24.37 | 26.89 | 76.00 | 15.64 |
| **July, 2021** | 34.50 | 23.79 | 24.47 | 89.68 | 310.20 |

**Source:** Meteorological Observatory, CSK HPKV, Regional Research Station, Dhaulakuan District Sirmour (HP) 173 031

**APPENDIX – II**

**Analysis of variance for design of experiment for summer season 2020**

| **Sr. No.** | **Source of Variation** | | **Mean Sum of Squares** | | | |
| --- | --- | --- | --- | --- | --- | --- |
|  |  |  | **Replications** | **Genotypes** | **Error** | **Fcal** |
|  | **Characteristics** | **df** | **2** | **19** | **38** |  |
| **1.** | Days to 50 per cent flowering |  | 2.4 | 125.97* | 3.98 | 31.66 |
| **2.** | Days to maturity (mature ripe stage) |  | 4.32 | 169.99* | 8.72 | 19.50 |
| **3.** | Plant height (cm) |  | 1.48 | 426.85* | 0.62 | 688.86 |
| **4.** | Number of ripe fruits per plant |  | 0.65 | 669.26* | 4.58 | 146.12 |
| **5.** | Average ripe fruit weight (g) |  | 0.03 | 2.39* | 0.02 | 145.12 |
| **6.** | Ripe fruit yield per plant (g) |  | 106.73 | 13293.28* | 106.34 | 125.01 |

**^*^**Significant at 5% level of significance

**Analysis of variance for design of experiment for winter season 2020**

| **Sr. No.** | **Source of Variation** |  | **Mean Sum of Squares** | | | |
| --- | --- | --- | --- | --- | --- | --- |
|  |  |  | **Replications** | **Genotypes** | **Error** | **Fcal** |
|  | **Characteristics** | **df** | **2** | **19** | **38** |  |
| **1.** | Days to 50 per cent flowering |  | 1.8 | 122.57* | 1.75 | 70.15 |
| **2.** | Days to maturity (mature ripe stage) |  | 2.82 | 47.15* | 1.17 | 40.39 |
| **3.** | Plant height (cm) |  | 3.89 | 473.12* | 3.17 | 149.38 |
| **4.** | Number of ripe fruits per plant |  | 0.14 | 688.28* | 1.05 | 657.96 |
| **5.** | Average ripe fruit weight (g) |  | 0.02 | 2.29* | 0.01 | 477.09 |
| **6.** | Ripe fruit yield per plant (g) |  | 45.94 | 11353.47* | 32.44 | 349.97 |

**^*^**Significant at 5% level of significance

**Analysis of variance for design of experiment for summer season 2021**

| **Sr. No.** | **Source of Variation** |  | **Mean Sum of Squares** | | | |
| --- | --- | --- | --- | --- | --- | --- |
|  |  |  | **Replications** | **Genotypes** | **Error** | **Fcal** |
|  | **Characteristics** | **df** | **2** | **19** | **38** |  |
| **1.** | Days to 50 per cent flowering |  | 3.2 | 115.73* | 2.52 | 46.00 |
| **2.** | Days to maturity (mature ripe stage) |  | 1.35 | 184.86* | 2.61 | 70.74 |
| **3.** | Plant height (cm) |  | 0.11 | 434.23* | 1.09 | 395.99 |
| **4.** | Number of ripe fruits per plant |  | 0.48 | 670.25* | 10.20 | 65.74 |
| **5.** | Average ripe fruit weight (g) |  | 0.04 | 2.39* | 0.02 | 142.59 |
| **6.** | Ripe fruit yield per plant (g) |  | 123.12 | 13358.89* | 113.44 | 117.76 |

**^*^**Significant at 5% level of significance

**Analysis of variance for design of experiment for pooled mean for summer seasons**

| **Sr. No.** | **Source of Variation** |  | **Mean Sum of Squares** | | | |
| --- | --- | --- | --- | --- | --- | --- |
|  |  |  | **Replication** | **Genotypes** | **Error** | **Fcal** |
|  | **Characters** | **df** | **2** | **20** |  |  |
| **1.** | Days to 50 per cent flowering |  | 1.4 | 120.01* | 2.26 | 53.21 |
| **2.** | Days to maturity (mature ripe stage) |  | 2.28 | 176.26* | 4.28 | 41.19 |
| **3.** | Plant height (cm) |  | 0.42 | 430.25* | 0.61 | 710.93 |
| **4.** | Number of ripe fruits per plant |  | 0.37 | 667.55* | 5.18 | 128.85 |
| **5.** | Average ripe fruit weight (g) |  | 0.03 | 2.39* | 0.02 | 146.58 |
| **6.** | Ripe fruit yield per plant (g) |  | 113.17 | 13307.62* | 81.88 | 162.52 |

**^*^**Significant at 5% level of significance
